# Intercellular transfer of mitochondrial DNA carrying metastasis-enhancing pathogenic mutations from high- to low-metastatic tumor cells and stromal cells via extracellular vesicles

**DOI:** 10.1186/s12860-021-00391-5

**Published:** 2021-10-07

**Authors:** Keizo Takenaga, Nobuko Koshikawa, Hiroki Nagase

**Affiliations:** Division of Cancer Genetics, Chiba Cancer Center Research Institute, Nitona, Chuoh-ku, Chiba, Japan

**Keywords:** Mitochondria, Mitochondrial DNA, Mutation, Extracellular vesicles, Intercellular transfer, Lung cancer, Tumor microenvironment, Metastasis

## Abstract

**Background:**

Mitochondrial DNA (mtDNA) carrying certain pathogenic mutations or single nucleotide variants (SNVs) enhances the invasion and metastasis of tumor cells, and some of these mutations are homoplasmic in tumor cells and even in tumor tissues. On the other hand, intercellular transfer of mitochondria and cellular components via extracellular vesicles (EVs) and tunneling nanotubes (TNTs) has recently attracted intense attention in terms of cell-to-cell communication in the tumor microenvironment. It remains unclear whether metastasis-enhancing pathogenic mutant mtDNA in tumor cells is intercellularly transferred between tumor cells and stromal cells. In this study, we investigated whether mtDNA with the NADH dehydrogenase subunit 6 (*ND6*) G13997A pathogenic mutation in highly metastatic cells can be horizontally transferred to low-metastatic cells and stromal cells in the tumor microenvironment.

**Results:**

When MitoTracker Deep Red-labeled high-metastatic Lewis lung carcinoma A11 cells carrying the *ND6* G13997A mtDNA mutation were cocultured with CellLight mitochondria-GFP-labeled low-metastatic P29 cells harboring wild-type mtDNA, bidirectional transfer of red- and green-colored vesicles, probably mitochondria-related EVs, was observed in a time-dependent manner. Similarly, intercellular transfer of mitochondria-related EVs occurred between A11 cells and α-smooth muscle actin (α-SMA)-positive cancer-associated fibroblasts (CAFs, WA-mFib), macrophages (RAW264.7) and cytotoxic T cells (CTLL-2). Intercellular transfer was suppressed by inhibitors of EV release. The large and small EV fractions (L-EV and S-EV, respectively) prepared from the conditioned medium by differential ultracentrifugation both were found to contain mtDNA, although only S-EVs were efficiently incorporated into the cells. Several subpopulations had evidence of LC3-II and contained degenerated mitochondrial components in the S-EV fraction, signaling to the existence of autophagy-related S-EVs. Interestingly, the S-EV fraction contained a MitoTracker-positive subpopulation, which was inhibited by the respiration inhibitor antimycin A, indicating the presence of mitochondria with membrane potential. It was also demonstrated that mtDNA was transferred into mtDNA-less ρ^0^ cells after coculture with the S-EV fraction. In syngeneic mouse subcutaneous tumors formed by a mixture of A11 and P29 cells, the mitochondria-related EVs released from A11 cells reached distantly positioned P29 cells and CAFs.

**Conclusions:**

These results suggest that metastasis-enhancing pathogenic mtDNA derived from metastatic tumor cells is transferred to low-metastatic tumor cells and stromal cells via S-EVs in vitro and in the tumor microenvironment, inferring a novel mechanism of enhancement of metastatic potential during tumor progression.

**Supplementary Information:**

The online version contains supplementary material available at 10.1186/s12860-021-00391-5.

## Background

We and others previously showed that certain mitochondrial DNA (mtDNA) somatic mutations or single nucleotide variants (SNVs) enhanced the invasion and metastasis of mouse and human cell lines, e.g., NADH dehydrogenase subunit 6 (*ND6)* G13997A in Lewis lung carcinoma cells [1, 2], *ND6* 13885insC in mouse fibrosarcoma cells [1, 2], *ND3* G10398A and *tRNA*^*Leu(CUN)*^ A12308G in human breast cancer cells [3, 4], *ATP6* T8993G and *ND3* A10398G in human prostate cancer cells [5, 6], *tRNA*^*Leu(CUN)*^ A3243T in human osteosarcoma cells [7] and 12S rRNA (*RNR1)* G709A in human hepatocellular carcinoma [8]. Furthermore, the frequency of predicted pathogenic mtDNA mutations was significantly correlated with distant metastasis in patients with non-small cell lung carcinoma (NSCLC) and colon cancers [2]. Although the mechanisms underlying the enhancement of metastasis have not been fully elucidated, it has been demonstrated that pathogenic mtDNA mutations or SNVs confer apoptotic resistance against various stresses to tumor cells [1, 3, 4, 9, 10] and increase the expression of various nuclear-encoded metastasis-related genes, such as antiapoptotic *Mcl-1*, *K-ras*, *c-myc*, and hypoxia-inducible factor-1α (*HIF-1α*), compared to their counterparts with wild-type mtDNA [1, 2]. Interestingly, although somatic mutations usually show heteroplasmy, some of the somatic mutations or SNVs in tumor cells and even in tumor tissues of a variety of tumors are homoplasmic [2, 11–15]. This phenomenon could be simply due to a low percentage of stromal cells that are supposed to have wild-type mtDNA in the tumor tissues analyzed, but it is possible that stromal cells also have the same mutant mtDNA as tumor cells. This possibility has never been tested.

Recent studies have highlighted intercellular communication between tumor cells and stromal cells in the tumor microenvironment via extracellular vesicles (EVs) and tunneling nanotubes (TNTs) [16–19]. EVs are mainly comprised of heterogeneous cell-derived membranous structures and across three subclasses: small EVs (S-EVs), large EVs (L-EVs) and an intermediate group known as small to large EVs [20]. Typical S-EVs are exosomes that are 50- to 150-nm, characterized by tetraspanins CD9, CD63 and CD81, and contain mitochondrial cargo and other cellular components. L-EVs include microvesicles that are 100- to 1000-nm, featured by a high content of annexin A1, and encapsulate mitochondria and other components [19, 20]. Autophagic EVs belong to the small to larger EV subclass and have the characteristics of expressing microtubule-associated protein 1 light chain 3 (LC3) [20]. In addition, mitovesicles are a recently identified novel population of EVs of mitochondrial origin that contain abundant mitochondrial markers and reflect the alteration of pathophysiological processes where mitochondrial dysfunction occurs and are much smaller than native mitochondria [21]. TNTs are actin-based direct cell-to-cell communication channels that serve to transfer a variety of molecules and organelles, including mitochondria and exosomes, which are thought to be the main delivery route to transfer intact mitochondria in solid tumors [18, 22, 23]. EV- and TNT-mediated cell-to-cell communication between cancer cells and stromal cells, such as bone marrow-derived mesenchymal stem cells, fibroblasts and endothelial cells, is reported to play roles in alterations in cancer cell bioenergetics, chemoresistance, invasion and metastasis [16–18, 22, 23]. However, because a variety of cellular components, such as cellular proteins, lipids, mRNAs and microRNAs, are transferred via EVs and TNTs from stromal cells to tumor cells and vice versa [19, 23], the exact functional roles of transferred mitochondria, particularly tumor-derived mutant mtDNAs, remain unknown. Furthermore, because mitochondria with pathogenic mutant mtDNAs are somewhat dysfunctional, they are considered to be subjected to degradation by mitophagy, a mitochondrial quality control mechanism [24]. At present, it is unclear whether intact circular pathogenic mutant mtDNAs are included in EVs and whether they are intercellularly transferred between tumor cells and stromal cells, including cancer-associated fibroblasts (CAFs), tumor-associated macrophages (TAMs) and immune cells, in the tumor microenvironment.

In the present study, we investigated whether mtDNAs harboring a metastasis-enhancing pathogenic mutation in highly metastatic cancer cells are transferred to low-metastatic cells and stromal cells in vitro and in the tumor microenvironment. The results showed that they are transferred to low-metastatic cells and stromal cells via certain subpopulations of S-EVs, indicating that selection and prevalence of transferred mtDNA during tumor growth and progression could enhance the metastatic ability of low-metastatic tumor cells and alter the phenotypes of stromal cells in the tumor microenvironment.

## Results

### Bidirectional intercellular transfer of mitochondria-related vesicles

**Fig. 1:**
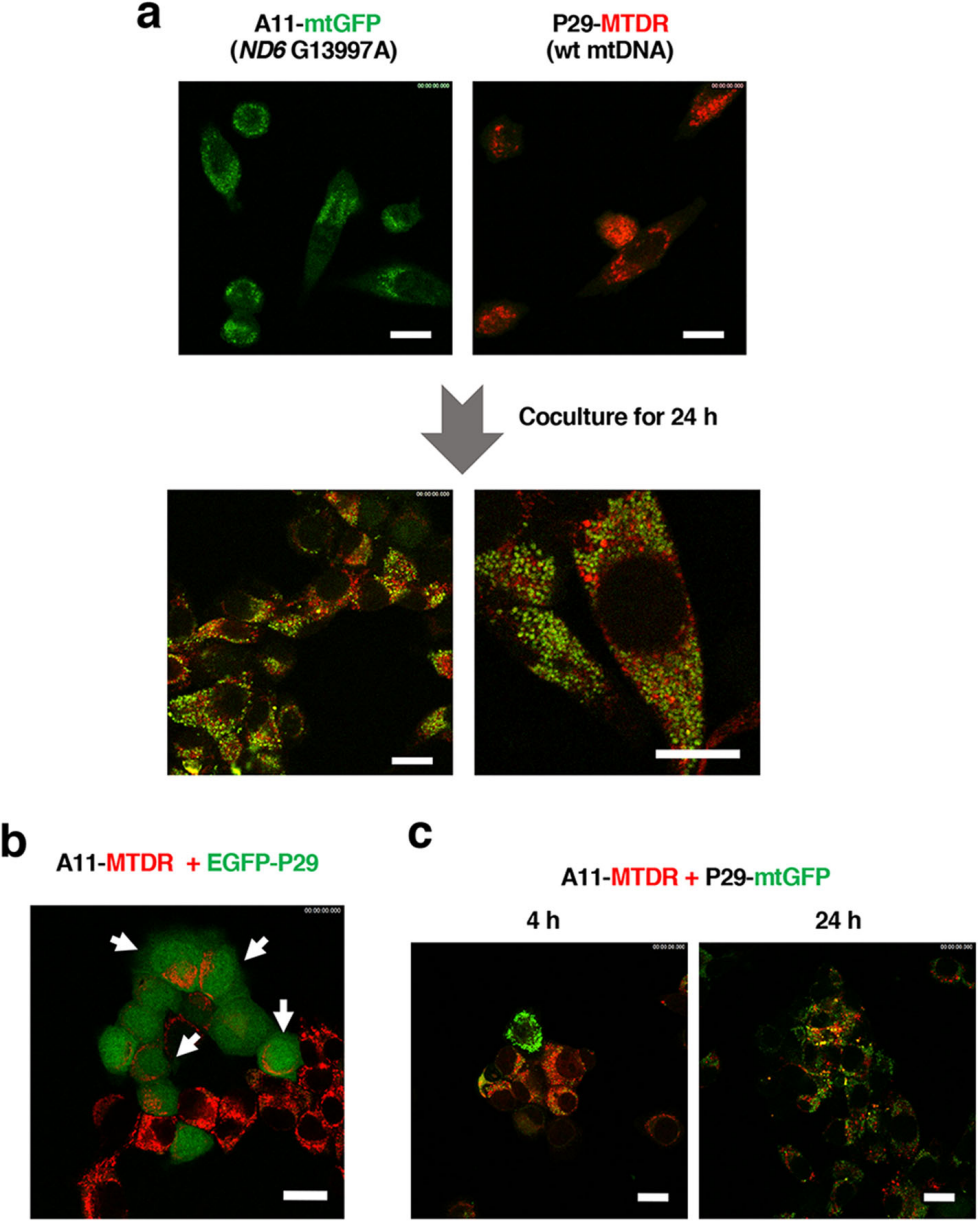
Intercellular transfer of mitochondria-related vesicles. (a) Intercellular transfer of mitochondria-related vesicles between A11 and P29 cells. mtGFP-labeled A11 cells (A11-mtGFP) and MTDR-labeled P29 cells (P29-MTDR) were cocultured for 24 h and observed under a laser confocal microscope. Scale bars: 20 μm. (b) Intercellular transfer of mitochondria-related vesicles of A11 cells to P29 cells expressing EGFP. MTDR-labeled A11 cells (A11-MTDR) were cocultured with EGFP-P29 cells for 24 h. Arrows indicate EGFP-P29 cells harboring red-colored mitochondrial-related vesicles. Scale bars: 20 μm. (c) Time course of intercellular transfer of mitochondria-related vesicles. MTDR-labeled A11 cells (A11-MTDR) were cocultured with mtGFP-labeled P29 cells (P29-mtGFP) for 4 h and 24 h. The cells were observed under a laser confocal microscope. Scale bars: 20 μm

**Fig. 2:**
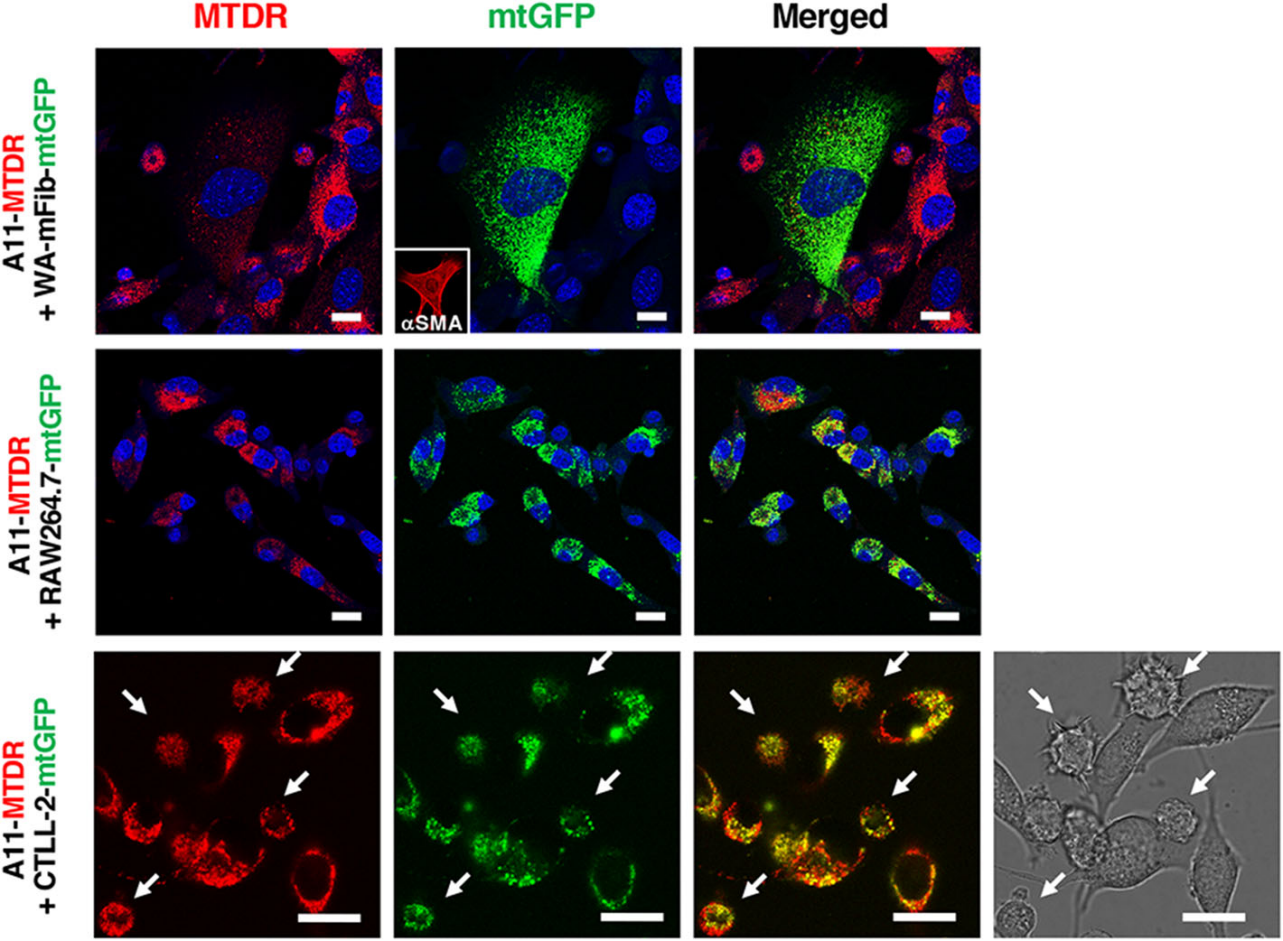
Intercellular transfer of mitochondria-related vesicles derived from A11 cells to CAFs, macrophages and cytotoxic T cells. MTDR-labeled A11 cells (A11-MTDR) were cocultured with mtGFP-labeled WA-mFib cells (the inset shows α-SMA immunostaining), RAW264.7 cells or CTLL-2 cells for 24 h. The cells were observed under a laser confocal microscope. Arrows in the bottom row indicate CTLL-2 cells. Scale bars: 20 μm

To examine whether mtDNA is intracellularly transferred, we labeled the mitochondria of mouse lung carcinoma P29 cells harboring wild-type mtDNA with MitoTracker Deep Red (P29-MTDR) and those of A11 cells carrying G13997A mtDNA with CellLight mitochondria-GFP (A11-mtGFP) and cocultured them for 24 h. As a consequence, we found that individual cells had both red- and green-colored vesicles within the cells, as assessed by confocal laser microscopy (Fig. 1a). The same outcome was obtained in cocultures of P29-mtGFP cells and A11-MTDR cells (Fig. S1). These results indicated that mitochondria or mitochondrial-related vesicles were transferred from A11 cells to P29 cells and vice versa. In addition, when EGFP-expressing P29 cells (EGFP-P29) were cocultured with A11-MTDR cells, red-colored vesicles were detected in the cytoplasm of noncontacting EGFP-P29 cells (Fig. 1b). Notably, the red-colored vesicles were unevenly localized in EGFP-P29 cells, excluding the possibility that the vesicles were merely attached to the outside of EGFP-P29 cells. Mitochondria-related vesicle transfer in the coculture of P29-mtGFP and A11-MTDR cells was detected as early as 4 h after the start of coculture (Fig. 1c). Furthermore, when A11-MTDR cells were cocultured with mtGFP-labeled α-smooth muscle actin (α-SMA)-positive CAFs (WA-mFib-mtGFP), macrophages (RAW264.7-mtGFP) or cytotoxic T cells (CTLL-2-mtGFP), we obtained similar results, although the degree of intercellular transfer was lower in the A11-WA-mFib coculture than in other cocultures (Fig. 2), perhaps due to the weak ability of WA-mFib cells to take up and release mitochondria-related vesicles. Bidirectional vesicle transfer was barely observed in A11 cells-normal fibroblasts (MEF) coculture (Fig. S2). Together, these results indicated that mitochondria or mitochondrial components were transferred not only between tumor cells but also between tumor cells and stromal cells.

### EVs contain mitochondrial components and mtDNA

**Fig. 3:**
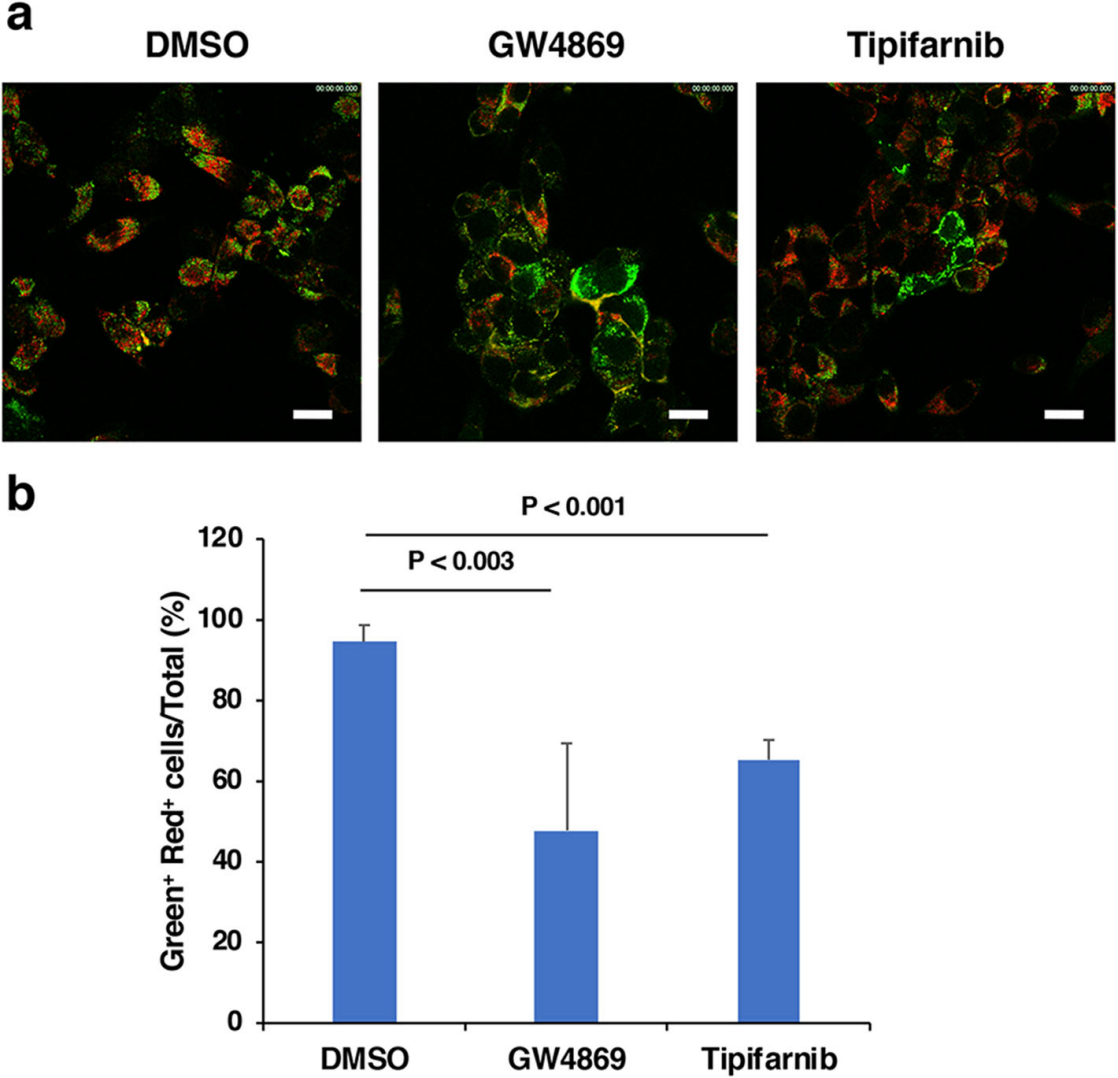
Effect of GW4869 and tipifarnib on intercellular transfer of mitochondria-related vesicles. (a) mtGFP-labeled A11 cells (A11-mtGFP) and MTDR-labeled P29 cells (P29-MTDR) were cocultured in the presence or absence of 10 μM GW4869 or 1 μM tipifarnib for 24 h and observed under a laser confocal microscope. Scale bars: 20 μm. (b) Semi-quantification of intercellular transfer of mitochondria-related vesicles. The number of green- and red-double positive cells per total number of cells in the images (*n* = 4, 5 and 6 images for DMSO, GW4869 and Tipifarnib group, respectively) was counted

**Fig. 4:**
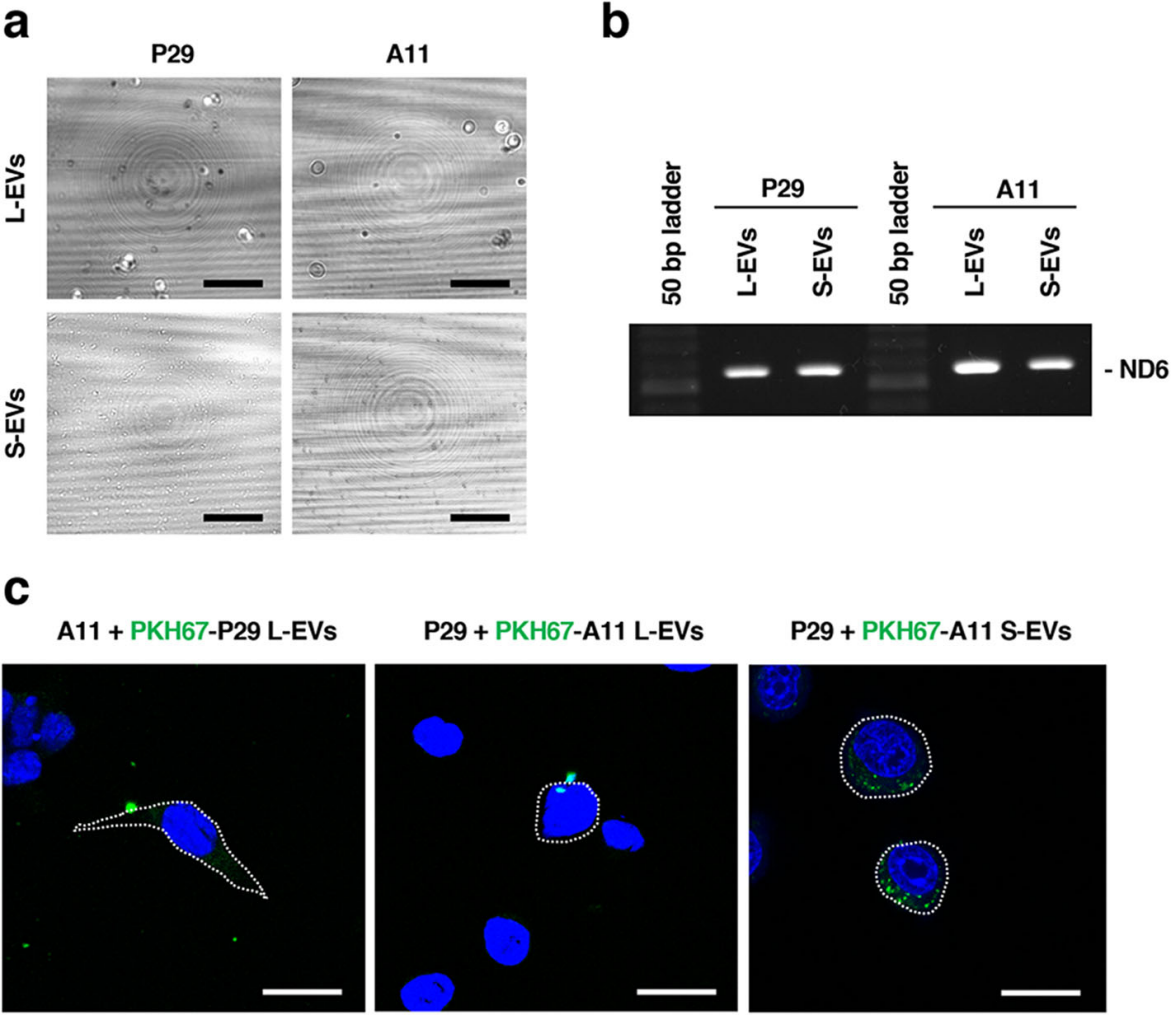
L-EVs and S-EVs isolated from the conditioned medium of P29 and A11 cells. (a) Reflection images of L-EVs and S-EVs. Scale bars: 10 μm. (b) PCR analysis of the presence of mtDNA *ND6* gene in L-EVs and S-EVs. (c) Localization of L-EVs and S-EVs added to P29 or A11 cells. P29 or A11 cells were incubated with PKH67-labeled L-EVs or S-EVs for 24 h and observed under a laser confocal microscope. Dashed lines represent the outline of the cells. Scale bars: 20 μm

**Fig. 5:**
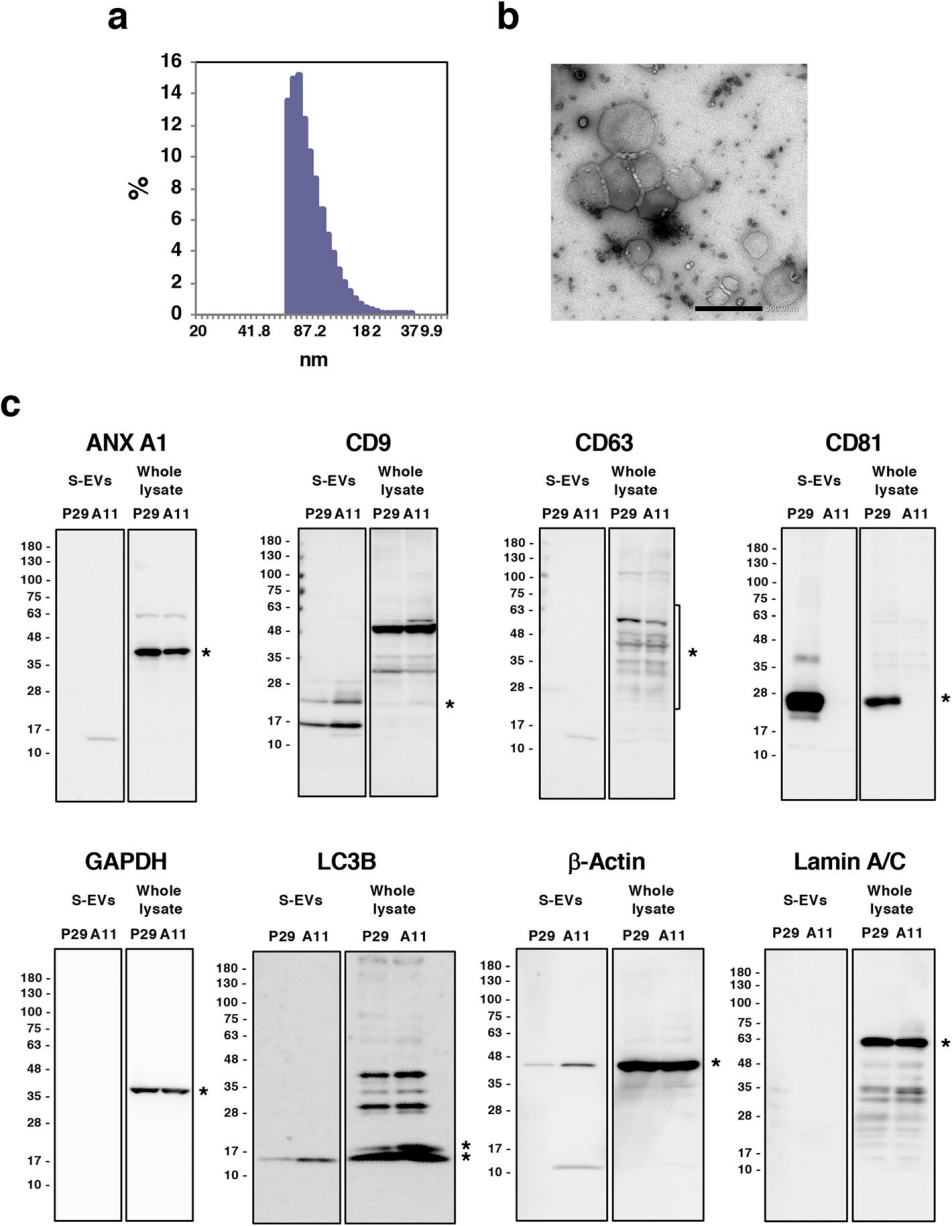
Characterization of S-EVs isolated from the conditioned medium of A11 cells. (a) A percent bar plot showing the distribution of particle diameter of A11 S-EVs. (b) Negative-stain TEM of A11 S-EVs. Bar: 500 nm. (c) Western blot analyses of various proteins in S-EVs isolated from the conditioned medium of P29 and A11 cells. P29 and A11 S-EVs (3 μg proteins) and whole cell lysates of P29 and A11 cells (30 μg proteins) were loaded on the same gel. Asterisks indicate the position of individual protein. In the LC3B immunoblot, the upper and lower asterisks indicate LC3-I and LC3-II, respectively. Uncropped Western blots images are shown in Fig. S7

**Fig. 6:**
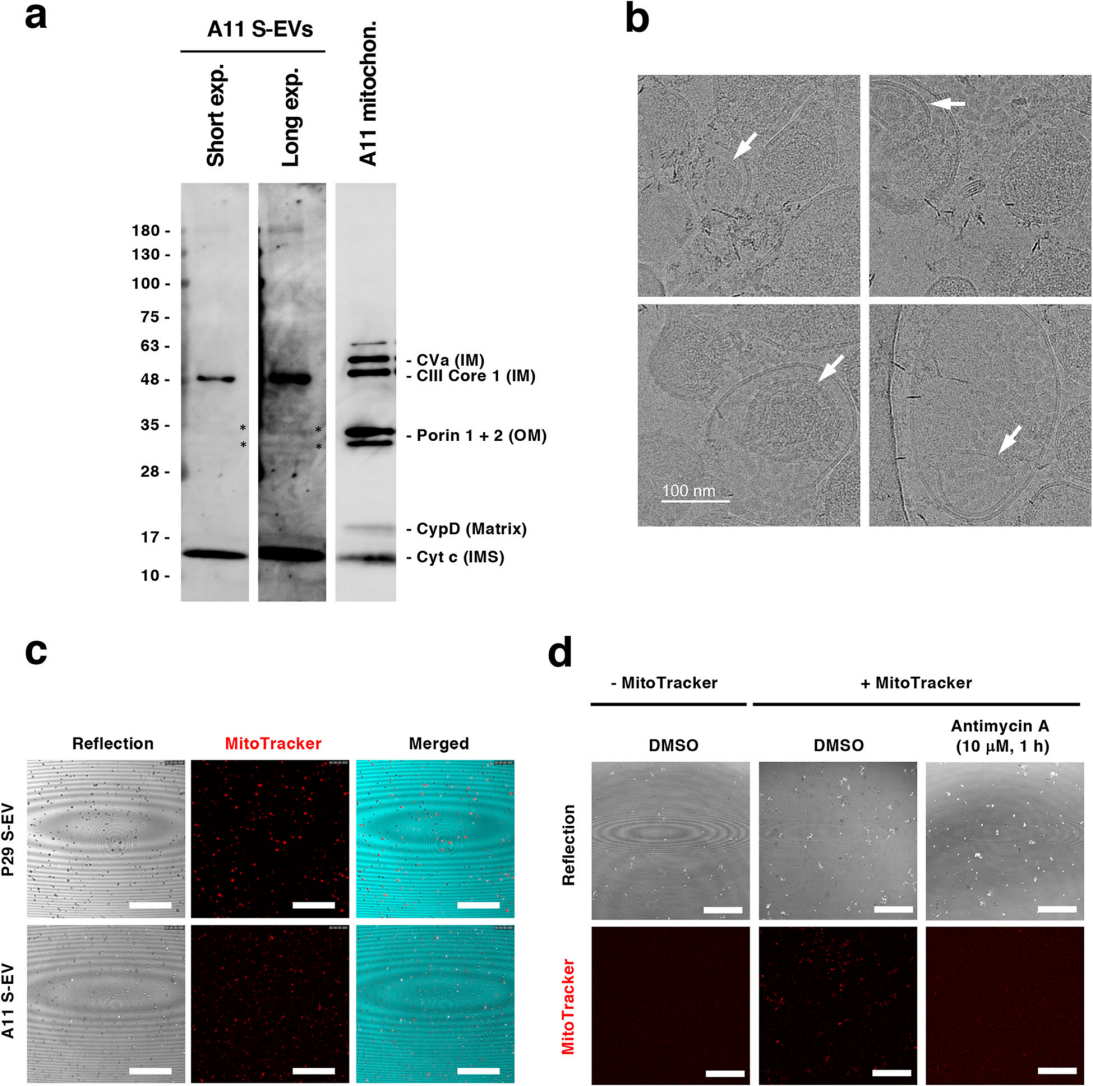
Mitochondrial components in A11 S-EVs. (a) Western blot analysis of mitochondrial membrane integrity in A11 S-EVs by using a cocktail of antibodies against mitochondrial proteins. CVa, CIII Core 1, Porin, CypD and Cyt c indicate inner membrane (IM) protein Complex Va, IM protein Complex III Core 1, outer membrane (OM) protein Porin, matrix space protein Cyclophilin D and intermembrane space (IMS) protein Cytochrome c, respectively. Lysates of A11 S-EVs and isolated A11 mitochondria were separated on the same gel. Images of short and long exposure of A11 S-EVs lane were shown. Asterisks show the positions of Porin 1 and 2. Uncropped Western blots images are shown in Fig. S8. (b) Cryo-TEM images of A11 S-EVs. Arrows indicate double membraned structures in S-EVs. Bar: 100 nm. (c) S-EVs isolated from the conditioned medium of MitoTracker Red-stained P29 and A11 cells. Scale bars: 10 μm. (d) Staining of A11 with MitoTracker Red. A11 S-EVs were stained with MitoTracker Red for 1 h in the presence or absence of 10 μM antimycin A. Note that aggregates of S-EVs were observed. Scale bars: 10 μm

To determine the mechanisms underlying intercellular mitochondria-related vesicle transfer, we carried out coculture of P29 and A11 cells in the presence or absence of the EV secretion inhibitors GW4869 and tipifarnib [25, 26]. The results showed that both inhibitors suppressed, although not completely, intercellular transfer (Fig. 3), suggesting the involvement of EVs released from the cells. Close observation of the coculture revealed the presence of both green- and red-colored vesicles in the areas where no cells existed (Fig. S3). Then, we prepared L-EV and S-EV fractions from A11 and P29 cell culture medium by differential ultracentrifugation (Fig. 4a). PCR analysis using *ND6* primers showed that both L-EV and S-EV fractions contained evidence of mtDNA (Fig. 4b); when these EV preparations were added to A11 or P29 cells, we found L-EVs to merely attached on the surface of cell membrane while cells actively incorporated S-EVs (Fig. 4c) in a manner that fairly resembled vesicle transfer images in the coculture (Fig. 1), prompting us to focus our investigation on S-EVs. A11 S-EVs showed a size range between approximately 70 and 300 nm (Fig. 5a) and showed the appearance of EVs, as demonstrated by negative-stain transmission electron microscopy (TEM) (Fig. 5b). The microvesicle marker protein annexin A1 in both P29 and A11 S-EV fractions (Fig. 5c) were absent in immunoblots. Among the classical exosome marker proteins, CD9 was detected in both P29 and A11 S-EV fractions, yet we found neither S-EV fraction had any enrichment of CD63; CD81 was observed in only the P29 S-EV fraction (Fig. 5c). Neither was glyceraldehyde-3-phosphate dehydrogenase (GAPDH), a protein abundant in the cytosol and the non-vesicular material in S-EV preparations [20]; the absence of GAPDH confirmed the lack of intracellular contaminants and non-vascular fractions in our findings. Of note, LC3, particularly LC3-II, was detected in both P29 and A11 S-EV fractions, indicating the presence of autophagy-related EV subpopulation [20]. The amount of LC3-II in P29 S-EV fraction was lower than the A11 S-EV fraction. We observed the presence of β-Actin in both S-EV fractions, while the protein had been reported to be rich in all types of EVs except exosomes [20] (Fig. 5c); the amount was relatively lower in P29 group. In A11 S-EV preparations, we detected traces of certain mitochondrial proteins, such as the inner membrane protein Complex III core 1 (CIII Core 1), the outer membrane proteins Porin 1 and 2, and the intermembrane protein Cytochrome c (Cyt c), although the relative abundance of individual protein markedly differed from that of intact mitochondria. The outer membrane protein Complex Va (CVa) and the matrix protein Cyclophilin D (CypD) were not seen (Fig. 6a). Cryo-TEM revealed double membrane and electron lucent structures in some population of A11 S-EVs (Fig. 6b); these double membrane structures were either mitochondrial membranes that lacked distinct cristae, or nuclear membranes. Since we detected mitochondrial membrane proteins but not nuclear lamins in the S-EV preparations (Fig. 5c), such double-membraned structures were more likely to be mitochondrial in origin. These results suggested that A11 S-EV fraction contained a subpopulation with degenerated mitochondrial fragments. Interestingly, however, some of the S-EVs isolated from the conditioned medium of MitoTracker Red-stained A11 and P29 cells emitted red fluorescence (Fig. 6c). Furthermore, when naïve A11 S-EVs were incubated with MitoTracker Red, some but not all S-EVs accumulated the dye, which was suppressed by antimycin A, an inhibitor of cellular respiration (Fig. 6d).

**Fig. 7:**
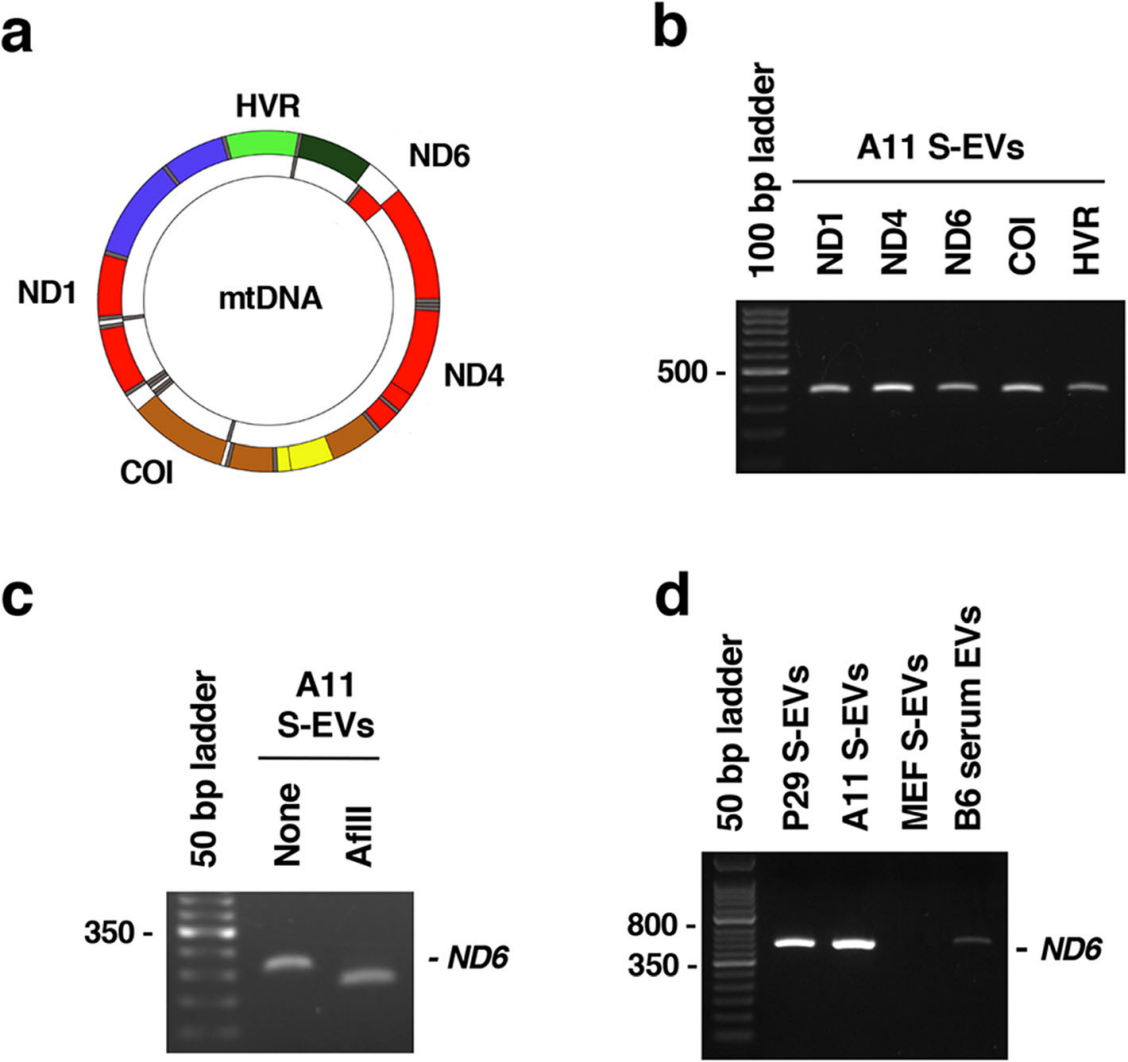
PCR analysis of the presence of mtDNA in S-EVs. (a) Location of the genes in mtDNA used for PCR analysis. (b) Presence of various mtDNA genes (*ND1*, *ND4*, *ND6*, *COI*, *HVR*) in A11 S-EVs. (c) PCR-RFLP analysis of the presence of mtDNA with the *ND6* G13997A mutation. The *ND6* gene 254 bp fragment amplified by PCR using mismatched primers was digested with AflII. The primers were designed to generate 223 bp and 31 bp fragments upon AflII digestion when the G13997A mutation was present. The smaller fragment was not visible. (d) Comparison of the amount of mtDNA in S-EVs isolated from the conditioned medium of P29 cells, A11 cells and MEFs and EVs in B6 serum. Equivalent amounts of EVs (~ 90 ng proteins) were used for PCR amplification of the *ND6* gene

We next examined whether full-length mtDNA existed in A11 S-EVs by PCR using primer pairs to amplify various locations of mtDNA [D-loop hypervariable region (*HVR)*, *ND1*, *COI*, *ND4* and *ND6*] (Fig. 7a). As a result, PCR analysis revealed the presence of all other genes in A11 S-EV preparation (Fig. 7b), suggesting the existence of full-length mtDNA. A11 S-EV preparation contained mtDNA with the G13997A mutation, as demonstrated by PCR-RFLP using mismatched primers that create the AflII restriction site (Fig. 7c). After isolating MEF S-EVs and EVs from normal C57BL/6 mouse serum using a commercially available exosome isolation reagent, we found the vesicles to contain a very small amount of mtDNA (Fig. 7d), indicating that normal cells could have ejected small amounts of mtDNA-containing EVs compared to tumor cells. For reference, we also provided data on the release of mtDNA-containing S-EVs from human cancer cell lines (HeLa, A549, DLD-1 and MIAPaCa-2) (Fig. S4).

### Transfer of mtDNA via S-EVs to ρ^0^ cells

**Fig. 8:**
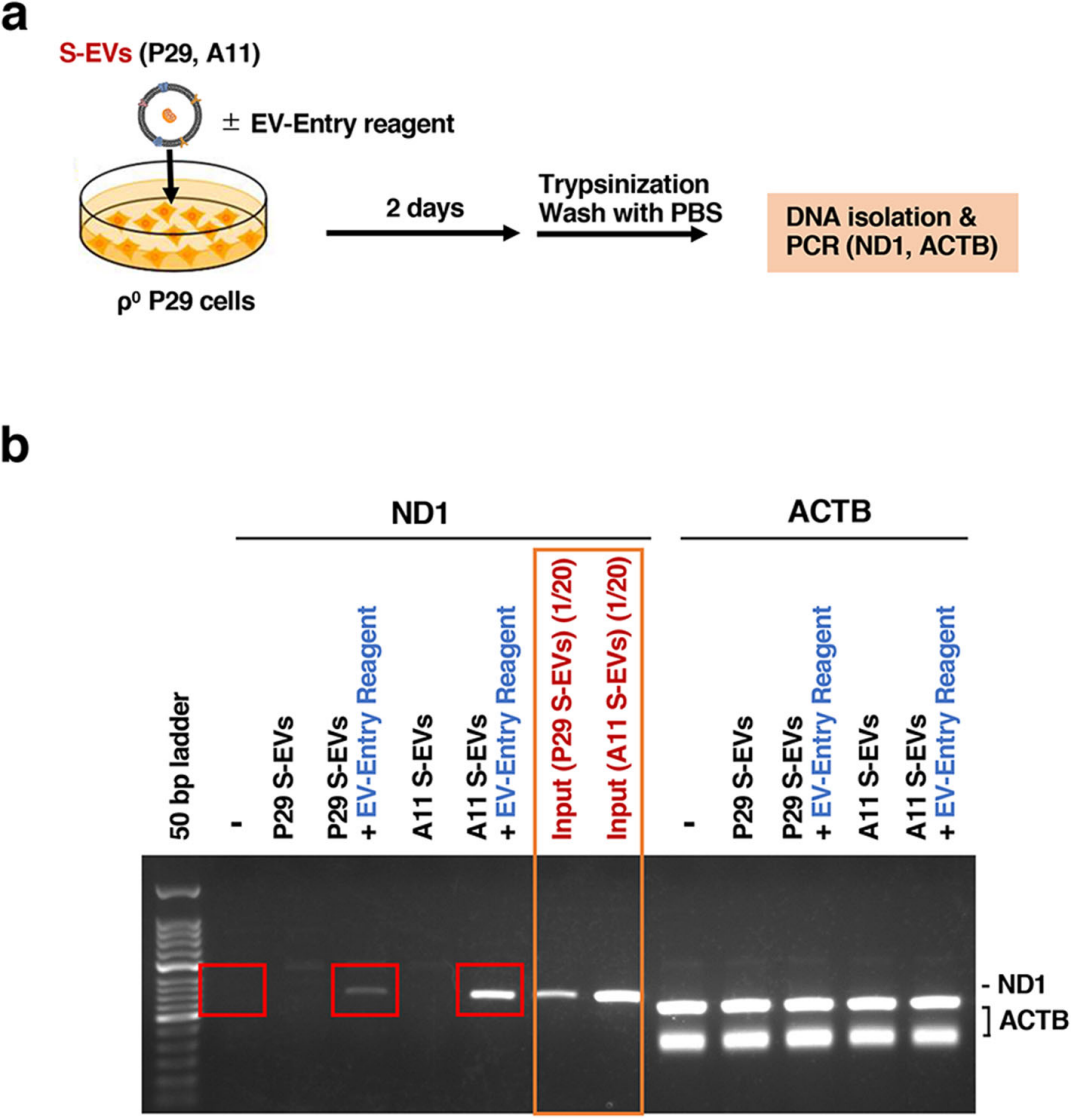
S-EV-mediated mtDNA transfer to ρ^0^P29 cells. (a) Schematic drawing of the procedure of S-EV-mediated transfer of mtDNA. S-EVs isolated from the conditioned media of P29 and A11 cells were incubated with ρ^0^P29 cells in the presence or absence of EV-Entry reagent for 2 days. The cells were detached from the dishes by trypsinization and washed extensively with PBS. (b) PCR analysis of mtDNA transfer to ρ^0^P29 cells via S-EVs. S-EVs-exposed ρ^0^P29 cell DNAs were isolated and subjected to PCR amplification of the *ND1* and *ACTB* genes. The PCR primers for *ACTB* gave rise to two bands. As inputs, one-twentieth of the amount of S-EVs added to ρ^0^P29 cells was also subjected to PCR analysis

When S-EVs prepared from the cell culture medium of A11-MTDR cells labeled with PHK67 (thus bicolor EVs) were added to P29 cells and cultured for 24 h, they were detected in the cells (Fig. S5). To verify whether mtDNA in S-EVs is actually transferred to recipient cells, we employed ρ^0^P29 cells because transferred mtDNA can be easily detected in these cells (Fig. 8a). We incubated S-EVs (equivalent to 2 μg protein) from P29 and A11 cells with ρ^0^P29 cells for 2 days in the presence or absence of the EV-Entry System. After incubation, the cells were treated with trypsin/EDTA for 10 min, washed extensively with PBS, and then subjected to DNA extraction. By PCR of the *ND1* gene, we detected mtDNA in ρ^0^P29 cells that were incubated with P29 S-EV and A11 S-EV fractions. However, we needed to use the EV-Entry System to clearly detect the transferred mtDNA (Fig. 8b), suggesting that ρ^0^P29 cells have a weak ability to incorporate exogenously added S-EVs. In contrast, for reference, when we incubated ρ^0^HeLa cells with HeLa S-EVs, we detected mtDNA in ρ^0^HeLa cells even in the absence of EV-Entry System (Fig. S6). These results indicate that mtDNA in S-EVs can be transferred to recipient cells in a cell-selective manner.

### Intercellular transfer of mitochondria-derived vesicles in the tumor microenvironment

**Fig. 9:**
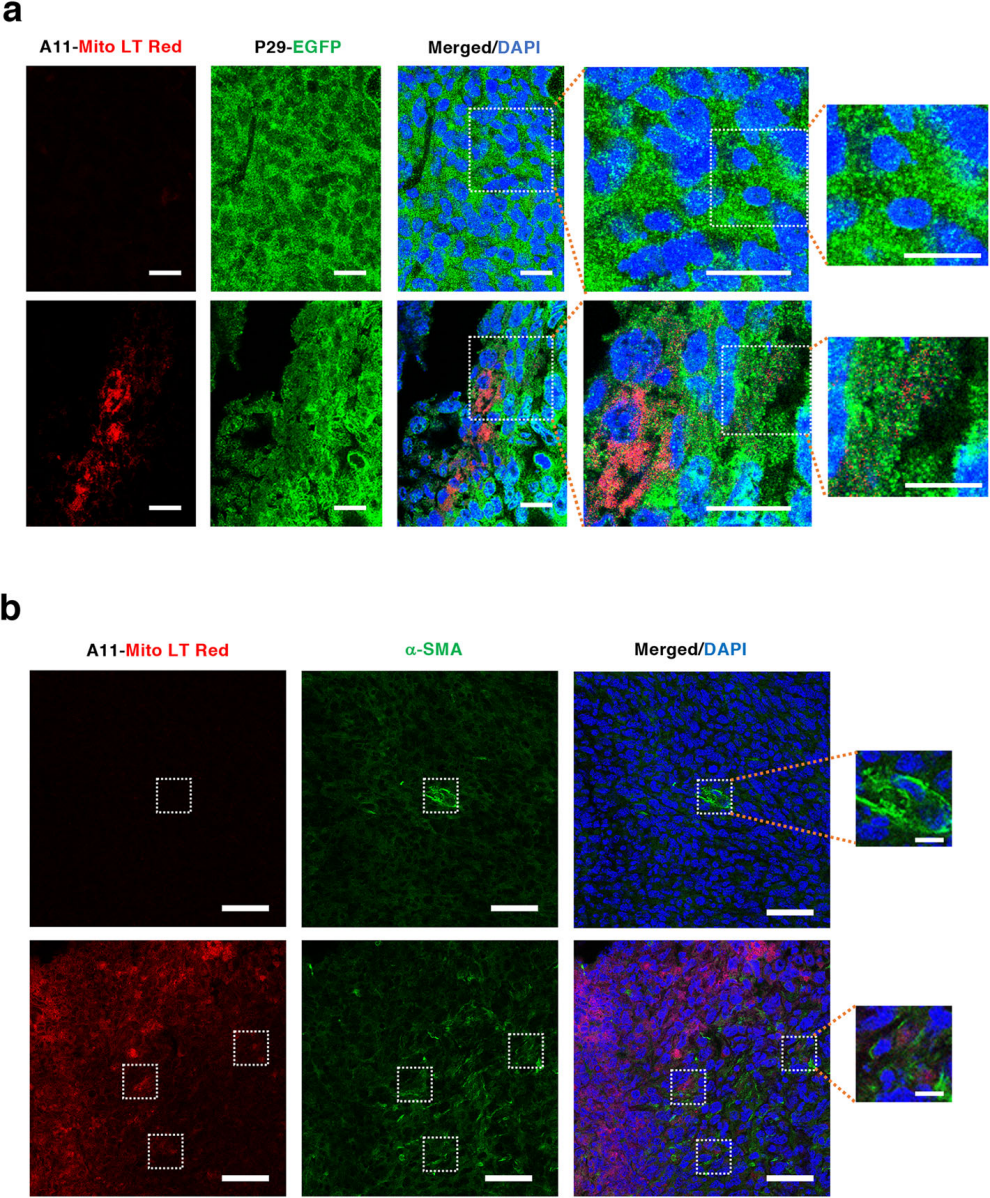
Diffusion of mitochondria-related vesicles of A11 cells in the homograft tumor microenvironment. (a) Transfer of mitochondria-related vesicles from MitoB LT Red-labeled A11 cells (A11-Mito LT Red) to P29-EGFP cells in a syngeneic tumor model. Upper panels. Area without A11-MitoB LT Red cells. Lower panels. Area with A11-MitoB LT Red cells. Note that red-colored vesicles diffused from A11-MitoB LT Red cells toward the upper right and localized within EGFP-P29 cells in the area surrounded by a dotted line. Scale bars: 10 μm and 20 μm for the rightmost panels and other panels, respectively. (b) Transfer of mitochondria-related vesicles from mitochondria of MitoB LT Red-labeled A11 cells (A11-MitoB LT Red) to CAFs. Upper panels. Area without A11-MitoB LT Red cells. Lower panels. Area with A11-Mito LT Red cells. Note that red-colored vesicles were released from A11-Mito LT Red cells and localized within α-SMA-positive CAFs in the areas surrounded by a dotted line. Scale bars: 10 μm and 50 μm for the rightmost panels and other panels, respectively

Finally, to investigate whether the observed cell-to-cell EV transfer occurs in the tumor microenvironment in a syngeneic mouse model, we intratumorally injected homografted MitoBright LT Red-labeled A11 cells (A11-Mito LT Red) into syngeneic EGFP-P29 tumors. Three days after the injection, tumors were resected and embedded in OCT compounds for preparing cryosections. Because of weak EGFP fluorescence, we immunostained cryosections (7-μm thick) with rabbit anti-GFP antibody followed by AF488-conjugated goat anti-rabbit IgG to clearly identify EGFP-P29 cells. Close examinations of the sections revealed that red-colored mitochondria-related vesicles were detected away from A11-Mito LT Red cells and localized in EGFP-P29 cells via a nonpassive transportation method (Fig. 9a, bottom, rightmost panel). In another experiment, we injected A11-Mito LT Red cells into P29 tumors. Immunostaining of cryosections for α-SMA showed red-colored vesicles in α-SMA-positive CAFs (Fig. 9b, bottom, dotted squares, rightmost panel). These results suggest the occurrence of intracellular mtDNA transfer in the tumor microenvironment, although the exact subtype of EVs involved in the transfer remained to be clarified.

## Discussion

The present results showed that deteriorated mitochondria containing metastasis-prone G13997A mtDNA of A11 cells was encapsulated in a certain subpopulation of S-EVs and released extracellularly. Several recent studies indicate that cells routinely release mitochondria extracellularly via EVs [27–29]; for instance, cardiomyocytes release subcellular LC3-positive “exophers” (3.5 ± 0.1 μm in size) in order to shuttle defective mitochondria for elimination [29]. The extracellular release of a particular form of naked mitochondria (~ 500 nm in size) is also said to be an alternative mitochondrial quality control mechanism [30]. The S-EVs in this study may be different from other S-EVs in the literature, for example recently identified mitovesicles that are both electron dense, and rich in mitochondrial proteins [21]; typical exosomes also do not contain double-stranded DNA [20]. Despite the lack of direct evidence, the process called “secretary autophagy” in which LC3-positive double membrane autophagosomes are released extracellularly without fusion with lysosomes [31], is likely the source of vesicles found in A11 S-EVs. Dysfunctional mitochondria are known to undergo mitophagy [24]. Because the G13997A mutation lowers Complex I activity, A11 mitochondria are likely to be susceptible to oxidative stress [1] and as a result prone to a greater amount of damage and degradation by mitophagy compared to those in P29 cells. It is likely that certain extracellularly released mitophagosomes can bypass fusion with lysosomes, as (1) A11 S-EVs preparation contain subpopulations harboring degenerated mitochondrial components, (2) P29 S-EV preparation contain a lower level of LC3-II than A11 S-EVs, and (3) P29 and MEF S-EVs contain lower quantities of mtDNA compared to A11 S-EVs; in this sense, we can refer to the process of extracellular release of degenerated mitochondria as “secretary mitophagy/autophagy”. Additionally, while some S-EV subpopulations still contained functioning mitochondria with detectable membrane potential, this was likely a reflection of transient remnants of respiratory activity from degenerated mitochondria in freshly prepared S-EVs. While one may question the physiological relevance of other cells incorporating wasted mitochondria, it is only presumable that mtDNA in such mitochondria still contained critical role, yet to be fully elucidated, inside the recipient cells.

This study demonstrated that G13997A mtDNA was horizontally transferred to P29 cells and stromal cells in vitro via S-EVs. Unfortunately, however, we still have not been able to demonstrate the physiological relevance of G13997A mtDNA transfer, i.e., changes in metastatic potential and apoptosis resistance of P29 cells, because the copy number of G13997A mtDNA did not substantially increase in P29 cells during approximately two months of culture after a single exposure of the cells to A11 S-EVs (data not shown). As in the cases of mitochondrial diseases, a critical threshold of the mutant/wild-type mtDNA ratio (heteroplasmy) must be exceeded before the pathogenic effect of the mutant mtDNA becomes apparent; the threshold is generally thought to be between 60 and 80% [32]. Therefore, we need to wait until the heteroplasmy level of G13997A mtDNA is high to investigate the consequence of mtDNA transfer, although no one knows how long is required. However, we believe that pathogenic mtDNA mutants become prevalent in tumor and stromal cells, either by positive selection or by chance [12, 14], which is supported by the fact that a pathogenic mtDNA somatic mutation that must have occurred in a mtDNA molecule among several hundreds of mtDNA molecules in a cell is often homoplasmic in tumor cells [2, 11–15]. Considering that tumor cells are continuously exposed to EVs released from other cells in the tumor microenvironment, we may need to expose P29 cells to A11 S-EVs several times in vitro to increase G13997A mtDNA copy number, thereby enabling the study of biological relevance of the G13997A mtDNA transfer. To our knowledge, the mtDNA mutation status in stromal cells in cancer tissues has never been investigated, and little is known about how it affects the activity of CAFs, TAMs and effector T cells. The prevalence of pathogenic mutant mtDNA derived from tumor cells in stromal cells may suppress mitochondrial respiration and results in a switch to a more glycolytic phenotype, which may cause CAFs to provide high-energy metabolites such as lactate to cancer cells [33] and stimulate T cell aging [34, 35]. These changes may enhance the invasion and metastasis of tumor cells. Thus, it is also important to examine the phenotypic changes of CAFs, TAMs and effector T cells caused by G13997A mtDNA transfer, which await investigation in the future. In addition, the mtDNA mutation status in the stroma in tumor tissues may need to be compared with that in tumor cells. Even though tumor-cell derived EVs have been reported to influence multiple aspects of cancer progression including survival, metastasis, angiogenesis and immune system [36], there are no reports that describe the direct effect of mutant mtDNAs in contrast to our present cell system, which may provide a valuable tool to investigate such issues in the future.

A limitation of the present study may be the absence of concrete evidence that mitochondrial components from A11 cells are actually transferred to P29 cells and CAFs in the tumor microenvironment. To address this, more sophisticated analyses, e.g., intravital microscopy and single cell analysis of the presence of mutant mtDNA in tumor or stromal cells distantly located from donor tumor cells may be required to demonstrate in vivo mtDNA transfer. Clarification of the exact type of EVs participating in the transfer is also critical, in addition to understanding the involvement of TNT-mediated mitochondrial transfer in this experimental setting.

Moreover, mtDNA mutations are known to accumulate during aging, and mitochondria become dysfunctional during aging [37]. Transfer of mutation-accumulated mtDNA in a senescent cell to surrounding cells may stimulate local senescence of tissues. Although we focused on mtDNA transfer in the tumor microenvironment, blocking mtDNA transfer could be a strategy to prevent not only tumor progression but also aging. Further studies are required to unveil the role of mutant mtDNA transfer in these processes.

## Conclusions

In this study, we demonstrated intercellular transfer of metastasis-enhancing mtDNA from high-metastatic lung cancer cells to low-metastatic cells and stromal cells such as CAFs, macrophages and cytotoxic T cells. Further studies will be focused on the consequence of mutant mtDNA transfer on the metastatic ability of low-metastatic cells, protumor activity of CAFs and TAMs and tumoricidal activity of cytotoxic T cells.

## Methods

### Reagents

GW4869, a neutral sphingomyelinase inhibitor, and tipifarnib, a farnesyl transferase inhibitor, were obtained from Cayman Chemical (Ann Arbor, MI, USA) and ChemScene LLC (Monmouth, NJ, USA), respectively. MitoTracker Red CMXRos, MitoTracker Deep Red FM (MTDR) and CellLight mitochondria-GFP (mtGFP) were supplied by Thermo Fisher Scientific (Waltham, MA, USA). MitoBright LT Red (MitoB LT Red) was purchased from Dojindo Co., Ltd. (Kumamoto, Japan).

### Cells and cell culture

High-metastatic A11 cells carrying the *ND6* G13997A mutation and low-metastatic P29 cells harboring wild-type mtDNA were established from Lewis lung carcinoma [1, 2, 10]. P29 cells expressing EGFP (EGFP-P29) were established by introduction of the pEGFP-N1 expression plasmid followed by G418 selection and cloning. WA-mFib cells, a mouse stromal cell line for human lung small cell carcinoma, and mouse RAW264.7 macrophages (RCB1925 and RCB0535, respectively, from RIKEN BioResource Research Center) [38] were cultured in Dulbecco’s modified Eagle’s medium (DMEM) supplemented with 10% FBS and 40 μg/ml gentamicin in a humidified atmosphere with 95% air/5% CO_2_ at 37 °C. CTLL-2 cells, a mouse cytotoxic T-cell line (RCB0637, RIKEN BioResource Research Center), were cultured in DMEM supplemented with 10% FBS and 50 ng/ml recombinant mouse IL-2 (BioLegend, San Diego, CA, USA). Human cervical cancer HeLa cells, human lung carcinoma A549 cells, human colon carcinoma DLD1 cells and human pancreatic carcinoma MIAPaCa-2 cells [39] were cultured in DMEM/10% FBS. MtDNA-less P29 (ρ^0^P29) cells [1] and ρ^0^HeLa cells (EB8 cells) [40] were cultured in DMEM supplemented with 0.1 mg/ml sodium pyruvate and 0.5 mg/ml uridine. Mouse embryonic fibroblasts (MEFs) were prepared and cultivated as described previously [41]. The addition of EVs to ρ^0^ P29 cells and ρ^0^HeLa cells was performed in the presence or absence of the EV-Entry System (System Biosciences, Palo Alto, CA, USA), which increases the rate of EV uptake and offloading of the cargo into the cytoplasm of the recipient cells.

### Labeling of mitochondria

For in vitro experiments, the cells and EVs were labeled with MTDR or mtGFP and MitoTracker Red, respectively. For in vivo experiments, cells were stained with MitoB LT Red, which is designed for mitochondrial retention for long-term visualization and shows stronger fluorescence signals than other commercially available dyes (Dojindo). Staining of the cells was performed according to the manufacturer’s instructions. The cells were observed under a Leica TSC SP8 confocal laser scanning microscope (Leica Microsystems, Wetzlar, Germany).

### Coculture

A11-MTDR or P29-MTDR cells (5 × 10^4^ cells) and P29-mtGFP, A11-mtGFP cells (5 × 10^4^ cells), respectively, were cocultured on glass coverslips in a well of a 12-well multiwell culture plate for 24 h. In other experiments, A11-MTDR cells (5 × 10^4^ cells) were cocultured with EGFP-P29 cells (5 × 10^4^ cells), WA-mFib-mtGFP cells (5 × 10^4^ cells), RAW264.7-mtGFP cells or MEF-mtGFP (5 × 10^4^ cells) for 24 h. The cells were fixed with 4% paraformaldehyde and observed under a confocal laser microscope. A11-MTDR cells (5 × 10^4^ cells) were also cocultured with CTLL-2-mtGFP cells (1 × 10^5^ cells) in a 35-mm glass-bottom culture dish for 24 h and observed under a confocal laser microscope without fixation.

### Preparation of EVs from cell culture media and normal mouse serum

EVs in cell culture medium were isolated by ultracentrifugation [42] with some modifications. Briefly, cells were cultured in DMEM containing 10% exosome-free FBS for 2 or 3 days. Exosome-free FBS was prepared by ultracentrifugation at 110,000×g overnight at 4 °C. The conditioned medium was centrifuged at 400×g for 10 min to remove cells, and the supernatant was centrifuged at 2000×g for 20 min to remove cell debris and apoptotic bodies. The resulting supernatant was ultracentrifuged at 15,000×g for 30 min at 4 °C; the pellet was washed with Dulbecco’s phosphate buffered saline (DPBS) and used as large EVs (L-EVs). The supernatant was passed through a Millex-GP filter (0.22 μm pore) (Merck-Millipore Burlington, MA, USA) and ultracentrifuged at 110,000×g for 90 min at 4 °C. The pellet was washed with DPBS to eliminate contaminated proteins and centrifuged again at 110,000×g for 90 min at 4 °C. The pellet was resuspended in DPBS and used as small EVs (S-EVs); EVs were sterilized by filtration through a Millex-GV filter (0.22 μm pore). EVs in C57BL/6 mouse serum were isolated using Total Exosome Isolation Reagent (from serum) (Thermo Fisher Scientific).

### Characterization of isolated S-EVs

The size range of the isolated S-EVs from the culture media of A11 cells was measured by a Zeta Potential/Particle Size Analyzer (Otsuka Electronics, Osaka, Japan). Annexin A1, CD9, CD63, CD81, GAPDH, LC3B, β-actin, Lamin A/C and mitochondrial proteins were detected by Western blot analyses using rabbit polyclonal anti-Annexin A1 antibody (Cell signaling Technology, Danvers, MA, USA), rabbit polyclonal anti-CD9 antibody (Flarebio Biotech LLC, Baltimore, MD, USA), rabbit polyclonal anti-CD63 antibody (Flarebio Biotech LLC), rabbit polyclonal anti-CD81 antibody (Cell signaling Technology), rabbit monoclonal anti-GAPDH antibody (Cell signaling Technology), rabbit polyclonal anti-LC3B antibody (Cell signaling Technology), mouse monoclonal anti-β-actin (Santa Cruz Biotechnology, Dallas, TX, USA), mouse monoclonal anti-Lamin A/C antibody (Santa Cruz Biotechnology) and Membrane Integrity WB Antibody Cocktail (abcam, Cambridge, UK). For this, EVs were dissolved in SDS-sample buffer, and the particle concentration was adjusted based on the protein concentration measured by the BCA method using bovine serum albumin (BSA) as a standard. For preparation of cell lysates of P29 and A11 cells, they were lysed in RIPA buffer containing cOmplete Protease Inhibitor Cocktail (Merck, Kenilworth, NJ, USA) and PhosSTOP (Merck Millipore, Billerica, MA, USA). The lysates were centrifuged at 10,000×g for 10 min at 4 °C, and the supernatants were used for immunoblot analysis. A11 mitochondria were isolated by using Mitochondria Isolation Kit for Cultured Cells (abcam). After SDS-polyacrylamide gel electrophoresis, the proteins were transferred to an Immobilon-P transfer membrane (Merck Millipore, Billerica, MA, USA). The membrane was incubated with washed with TBS-T (5 mM Tris/13.8 mM NaCl/0.05% Tween-20), and then incubated with horseradish peroxidase (HRP)-conjugated goat anti-rabbit IgG or goat anti-mouse IgG. Proteins were detected using ECL Plus Western blotting detection reagent (Amersham Biosciences, Piscataway, NJ, USA). For staining of EVs with PKH67, isolated L-EVs and S-EVs were stained using PKH67 Fluorescence Cell Linker Kits (Sigma-Aldrich, St. Louis, MO, USA) for 5 min, resuspended in DPBS, and centrifuged again at 15,000×g for 30 min and 110,000×g for 90 min, respectively, at 4 °C. The pellet was resuspended in DPBS.

### Transmission electron microscopy (TEM)

Negative-stain TEM and cryo-TEM of A11 S-EVs were performed by JEOL Ltd. (Tokyo, Japan).

### Detection of mtDNA in EVs and ρ^0^ cells by polymerase chain reaction (PCR)

For detection of mtDNA in EVs, the EVs were dissolved in 20 mM NaOH, incubated for 10 min at 95 °C, neutralized with 1 M Tris-HCl (pH 8.0), and then subjected to PCR analysis. For detection in mtDNA in ρ^0^P29 cells, the cells were detached from culture dishes with trypsin-EDTA (Sigma-Aldrich, Co., LLC, Saint Louis, MO, USA) at 37 °C for 10 min, washed extensively with DPBS three times, and then dissolved in DirectPCR Lysis Reagent (Cell) (Viagen Biotech, Inc., Los Angeles, CA, USA) containing 0.2 mg/ml proteinase K. After incubation at 55 °C for 60 min with shaking, proteinase K was inactivated by heating at 85 °C for 45 min. PCR was performed using GoTaq Hot Start DNA polymerase (Promega Corp., Madison, WI, USA). The PCR conditions were 94 °C for 5 min followed by 32 cycles of 94 °C for 30 s, 58 °C for 30 s, and 72 °C for 30 s. The primers used were 5′-CCGGGCCCATTAAACTTGGG-3′ and 5′- TAGTGTTTTTGGGGTTTGGCATT-3′ for *HVR*, 5′-CTAGCAGAAACAAACCGGGC-3′ and 5′-ATGGTGGTACTCCCGCTGTA-3′ for *ND1*, 5′-GACTTGCAACCCTACACGGA − 3′ and 5′-TGTGGTGTAAGCATCTGGGT-3′ for *COI*, 5′-ACCACTAACCTGACTATCAAGCC-3′ and 5′-GTTTGGTTCCCTCATCGGGT-3′ for *ND4*, 5′-GTTGGTTGTCTTGGGTTAGCAT-3″ and 5′-CTACCCCAATCCCTCCTTCCA-3′ for *ND6*, and 5′-CTCTGGCTCCTAGCACCATGAAGA-3′ and 5′-GTAAAACGCAGCTCAGTAACAGTCCG-3′ for *ACTB*. Aliquots of the PCR products were electrophoresed in 1.5% agarose gels and visualized on a transilluminator after staining with ethidium bromide. For recognition of the G13997A mutation by PCR-RFLP, a 254 bp fragment containing the 13,997 G > A site was amplified by PCR using the mismatched primers forward primer: 5′-CCCACTAACAATTAAACCTAAACCTCCATActTA-3′, (small letters indicate the mismatch site) and reverse primer: 5′-GGGGCAGGTAGGTCAATGAA-3′ to create a restriction site for AflII [1]. After digestion with AflII, the restriction fragments (223 bp and 31 bp) were separated in a 3% agarose gel. For detection of mtDNA in EVs from human cancer cells, the following primers were used: 5′- TCTTTCATGGGGAAGCAGAT-3′ and 5′- GCACTCTTGTGCGGGATATT-3′ for *HVR*, 5′- TCATGACCCTTGGCCATAAT-3′ and 5′- GGGGAATGCTGGAGATTGTA-3′ for *ND1*, 5′- ACGTTGTAGCCCACTTCCAC-3′ and 5′- GGGTTCTTCGAATGTGTGGT-3′ for *COI*, 5′-TGAACGCAGGCACATACTTC-3′ and 5′-TGTTTGTCGTAGGCAGATG-3′ for *ND4*, and 5′- CCCCGAGCAATCTCAATTAC-3′ and 5′-GGTGTGGTCGGGTGTGTTAT-3′ for *ND6*. For the detection of mtDNA in ρ^0^HeLa cells, the following primers were used: 5′- GTTGGTTGTCTTGGGTTAGCAT-3′ and 5′- CTACCCCAATCCCTCCTTCCA-3′ for *ND6*, and 5′-TGACGGGGTCACCCACACTGTGCCCATCTA-3′ and 5′- CTAGAAGCATTTGCGGTGGACGATGGAGGG-3′ for *ACTB*.

### Animal experiments

Five- to six-week-old male C57BL/6 J mice (CLEA Japan, Osaka, Japan) were used in this study. The mice were housed in a barrier facility under specific pathogen-free conditions and assessed with regard to health every day. For the in vivo mitochondrial distribution in tumor tissues, syngeneic transplantable P29 cells or EGFP-P29 cells (1 × 10^6^ cells) were subcutaneously implanted into the mice. When the tumor size reached approximately 0.75 cm^3^, another syngeneic A11 cell line (5 × 10^6^ cells) labeled with MitoB LT Red was injected intratumorally. After 3 days, homograft tumors were excised and immediately embedded and frozen in OCT compound for subsequent analysis. The mice were euthanized by CO_2_ inhalation at the end of the study.

### Immunostaining

Cryostat sections (7 μm thick) of homograft tumor tissues containing EGFP-P29 and MitoB LT Red-labeled A11 cells were fixed with 4% paraformaldehyde and stained with rabbit polyclonal anti-GFP antibody (Proteintech, Sankt Leon-Rot, Germany) followed by Alexa Fluor 488 (AF488)-conjugated anti-rabbit IgG. In this case, to clearly visualize EGFP-P29 cells, immunostaining with anti-GFP antibody was employed. Cryostat sections of tumor tissues containing P29 and MitoB LT Red-labeled A11 cells were fixed with 4% paraformaldehyde for 10 min and blocked with 1% BSA in DPBS. The sections were treated with an M. O. M. Immunodetection Kit (Vector Laboratories, Burlingame, CA, USA) and then incubated with mouse monoclonal anti-α-SMA (clone 1A4) (Dako, Jena, Germany) antibody followed by AF488-conjugated goat anti-mouse IgG. The sections were counterstained with DAPI and observed under a Leica TSC SP8 confocal laser scanning microscope. WA-mFib cells were also immunostained with the anti-α-SMA antibody followed by AF594-conjugated goat anti-mouse IgG.

## Supplementary Information

**Additional file 1 Fig. S1.** Intercellular transfer of mitochondria-related vesicles between A11 and P29 cells. mtGFP-labeled P29 cells (P29-mtGFP) and MTDR-labeled A11 cells (A11-MTDR) were cocultured for 24 h. Scale bars: 20 μm. **Fig. S2.** Intercellular transfer of mitochondria-related vesicles between A11 cells and MEF cells. MTDR-labeled A11 cells (A11-MTDR) and mtGFP-labeled MEF cells (MEF-mtGFP) were cocultured for 24 h. Scale bars: 20 μm. **Fig. S3.** Red- and green-colored mitochondria-related vesicles in the coculture of A11-MTDR and P29-mtGFP, WA-mFib-mtGFP or RAW264.7-mtGFP. Note that red- and green-colored mitochondriarelated vesicles exist in the areas where no cells are present. Scale bars: 30 μm. **Fig. S4.** PCR analysis of the presence of mtDNA in S-EVs isolated from various human cancer cell lines. S-EVs were isolated from the conditioned medium of HeLa cells, A549 cells, DLD1 cells and MIAPaCa-2 cells and subjected to PCR amplification of various mtDNA genes. **Fig. S5.** Incorporation of A11 S-EVs into P29 cells. S-EVs isolated from the conditioned media of A11-MTDR cells were stained with PKH67, resulting in red and green two-colored PKH-67-positive S-EVs. They were then added to P29 cells and incubated for 24 h. Note that two-colored S-EVs are within P29 cells. Scale bars: 20 μm. **Fig. S6.** S-EV-mediated mtDNA transfer to ρ^0^HeLa cells. S-EVs isolated from the conditioned media of HeLa cells were incubated with ρ^0^HeLa cells in the presence or absence of EV-Entry reagent for 2 days. The cells were detached from the dishes by trypsinization and washed extensively with PBS. DNA was isolated and then subjected to PCR amplification of *ND1* and *ACTB* (*β*-actin). The primers for *ND1* were the same as those described in Fig. S4. The primers for *ACTB* were used as a loading control. As an input, one-twentieth of the amount of S-EVs added to ρ^0^HeLa cells was also subjected to PCR analysis. **Fig. S7**. Full-size images of Western blots. **Fig. S8**. Full-size images of Western blots. The yellow dotted line indicates the cropped region.

## Data Availability

All data generated or analyzed during this study are included in this published article and its Additional files.

## Abbreviations

ACTB: β-Actin
AF: Alexa Fluor
α-SMA: α-Smooth muscle actin
BSA: Bovine serum albumin
CAF: Cancer-associated fibroblast
DMEM: Dulbecco’s modified Eagle’s medium
DPBS: Dulbecco’s phosphate-buffered saline
EGFP: Enhanced green fluorescent protein
EV: Extracellular vesicle
FBS: Fetal bovine serum
HVR: D-loop hypervariable region
L-EVs: Large extracellular vesicles
MEF: Mouse embryonic fibroblast
MitoB LT Red: MitoBright LT Red
mtDNA: Mitochondrial DNA
mtGFP: CellLight mitochondria-GFP
MTDR: MitoTracker Deep Red
NSCLC: Non-small cell lung carcinoma
ND: NADH dehydrogenase subunit
PCR: Polymerase chain reaction
RFLP: Restriction fragment length polymorphism
S-EVs: Small extracellular vesicles
TAM: Tumor-associated macrophage
TEM: Transmission electron microscopy
TNT: Tunneling nanotube
