# Supplementary material for: Intercellular transfer of mitochondrial DNA carrying metastasis-enhancing pathogenic mutations from high- to low-metastatic tumor cells and stromal cells via extracellular vesicles
Source: BMC Mol Cell Biol. 2021 Oct 7;22:52. doi: 10.1186/s12860-021-00391-5 (PMC8496074; doi:10.1186/s12860-021-00391-5)
Supplement: Supplementary file 1 — Additional file 1 Fig. S1. Intercellular transfer of mitochondria-related vesicles between A11 and P29 cells. mtGFP-labeled P29 cells (P29-mtGFP) and MTDR-labeled A11 cells (A11-MTDR) were cocultured for 24 h. Scale bars: 20 μm. Fig. S2. Intercellular transfer of mitochondria-related vesicles between A11 cells and MEF cells. MTDR-labeled A11 cells (A11-MTDR) and mtGFP-labeled MEF cells (MEF-mtGFP) were cocultured for 24 h. Scale bars: 20 μm. Fig. S3. Red- and green-colored mitochondria-related vesicles in the coculture of A11-MTDR and P29-mtGFP, WA-mFib-mtGFP or RAW264.7-mtGFP. Note that red- and green-colored mitochondriarelated vesicles exist in the areas where no cells are present. Scale bars: 30 μm. Fig. S4. PCR analysis of the presence of mtDNA in S-EVs isolated from various human cancer cell lines. S-EVs were isolated from the conditioned medium of HeLa cells, A549 cells, DLD1 cells and MIAPaCa-2 cells and subjected to PCR amplification of various mtDNA genes. Fig. S5. Incorporation of A11 S-EVs into P29 cells. S-EVs isolated from the conditioned media of A11-MTDR cells were stained with PKH67, resulting in red and green two-colored PKH-67-positive S-EVs. They were then added to P29 cells and incubated for 24 h. Note that two-colored S-EVs are within P29 cells. Scale bars: 20 μm. Fig. S6. S-EV-mediated mtDNA transfer to ρ0HeLa cells. S-EVs isolated from the conditioned media of HeLa cells were incubated with ρ0HeLa cells in the presence or absence of EV-Entry reagent for 2 days. The cells were detached from the dishes by trypsinization and washed extensively with PBS. DNA was isolated and then subjected to PCR amplification of ND1 and ACTB (β-actin). The primers for ND1 were the same as those described in Fig. S4. The primers for ACTB were used as a loading control. As an input, one-twentieth of the amount of S-EVs added to ρ0HeLa cells was also subjected to PCR analysis. Fig. S7. Full-size images of Western blots. Fig. S8. Full-size images of Western blots. T [file 12860_2021_391_MOESM1_ESM.pdf]

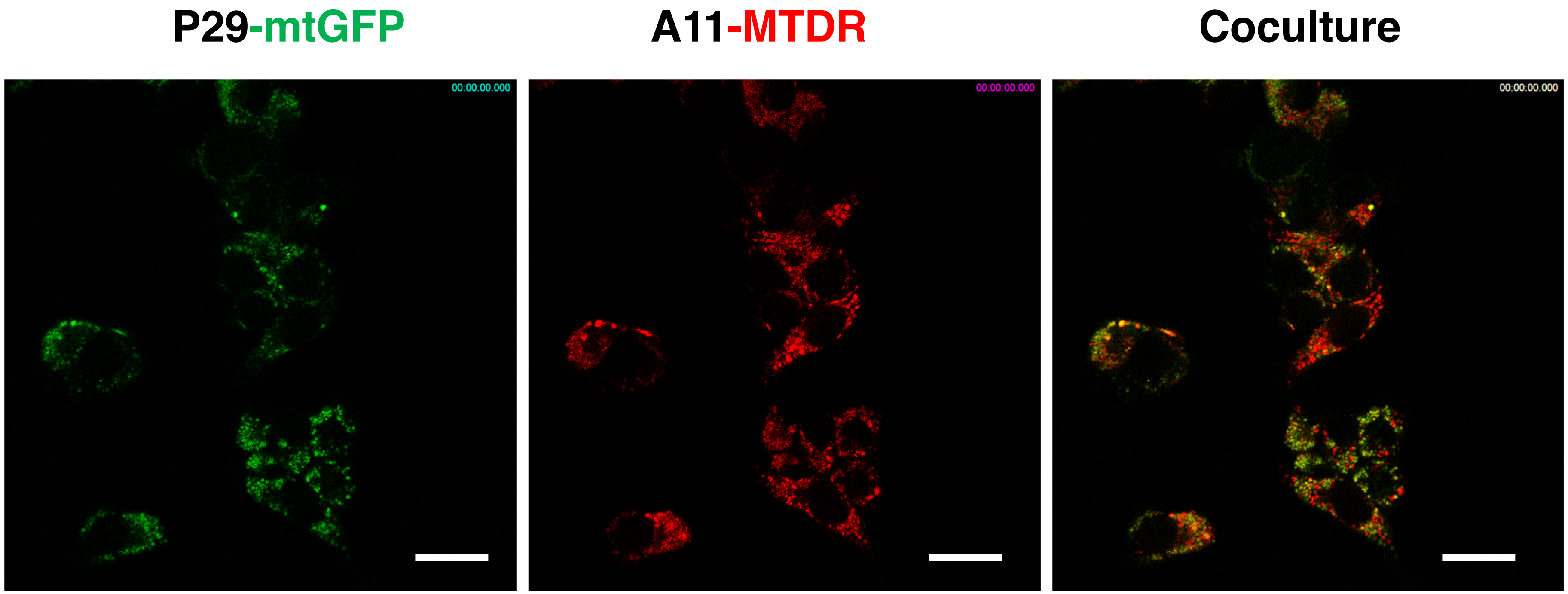

Fig. S1. Intercellular transfer of mitochondria-related vesicles between A11 and P29 cells. mtGFP-labeled P29 cells (P29-mtGFP) and MTDR-labeled A11 cells (A11-MTDR) were cocultured for 24 h. Scale bars: 20 μm.

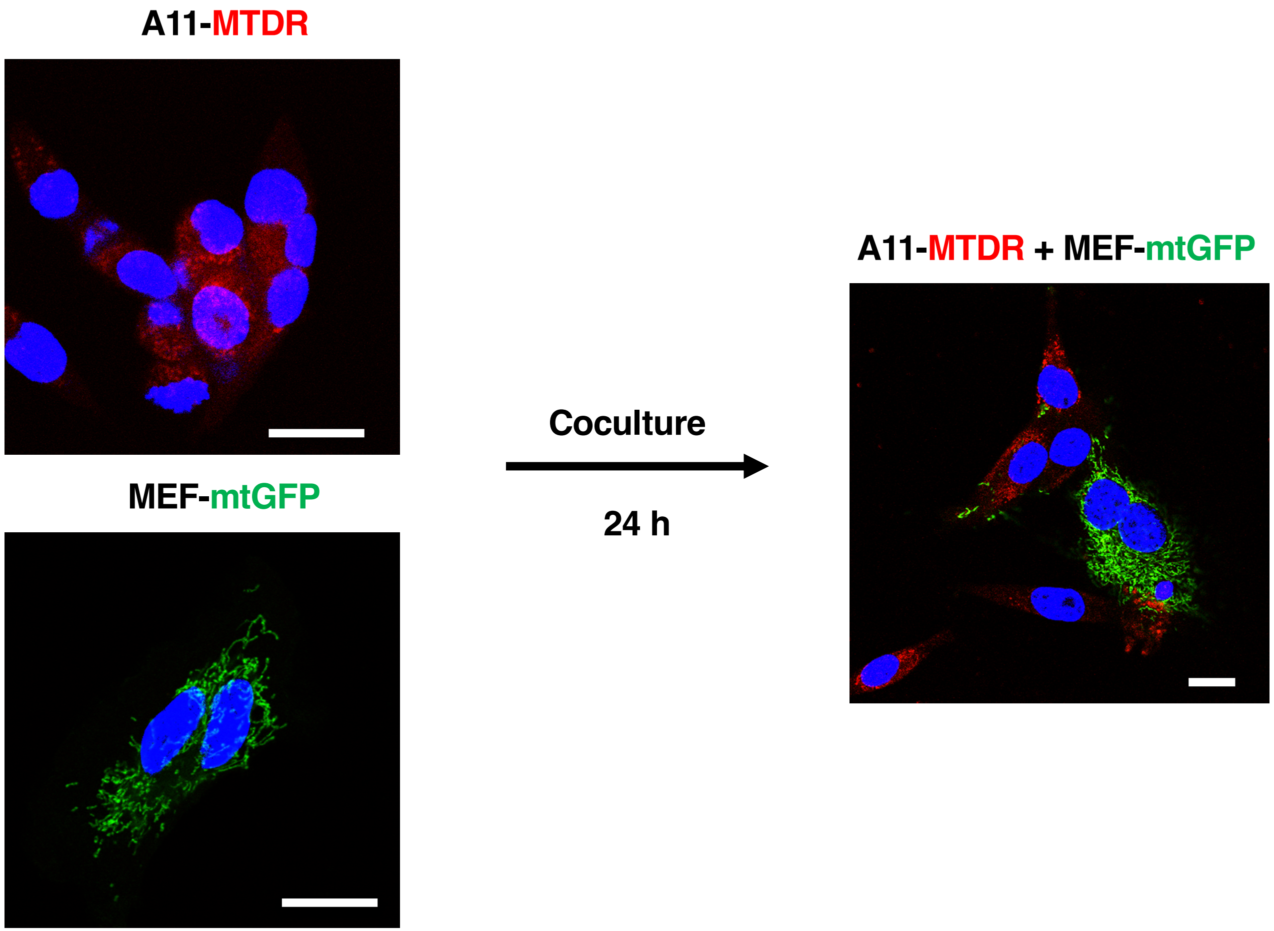

Fig. S2. Intercellular transfer of mitochondria-related vesicles between A11 cells and MEF cells. MTDR-labeled A11 cells (A11-MTDR) and mtGFP-labeled MEF cells (MEF-mtGFP) were cocultured for 24 h. Scale bars: 20 μm.

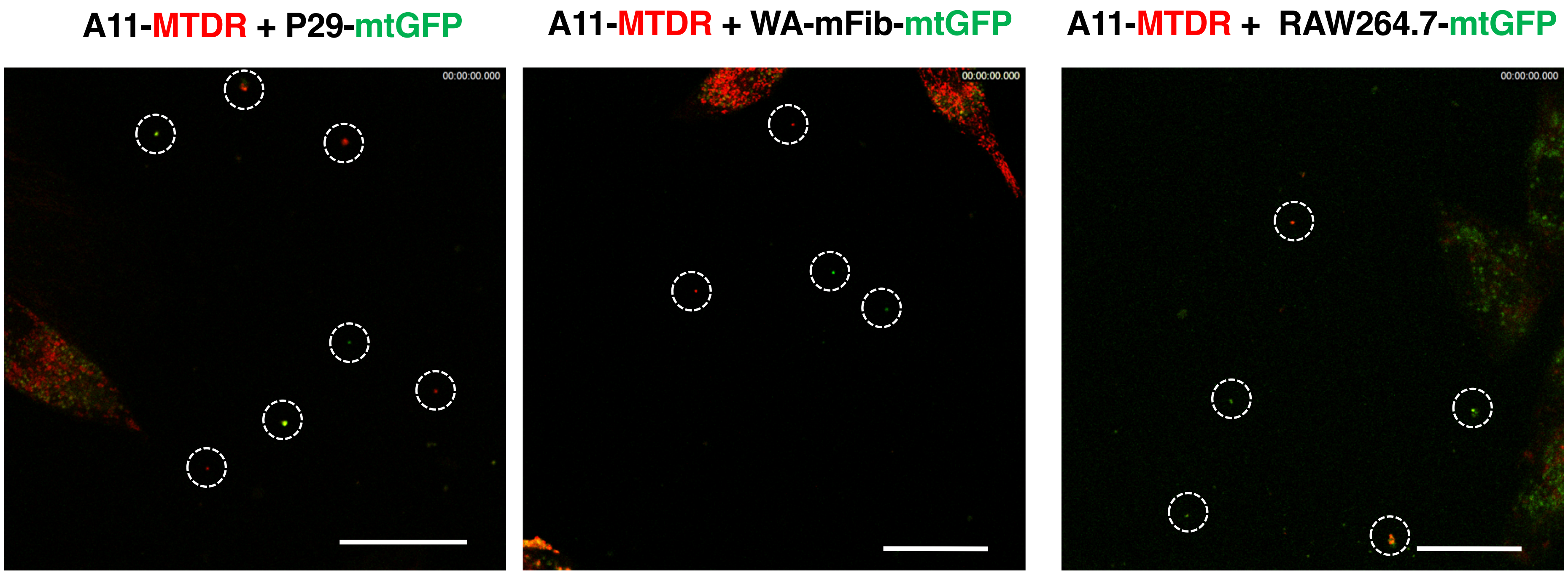

Fig. S3. Red- and green-colored mitochondria-related vesicles in the coculture of A11-MTDR and P29-mtGFP, WA-mFib-mtGFP or RAW264.7-mtGFP. Note that red- and green-colored mitochondria-related vesicles exist in the areas where no cells are present. Scale bars: 30 μm.

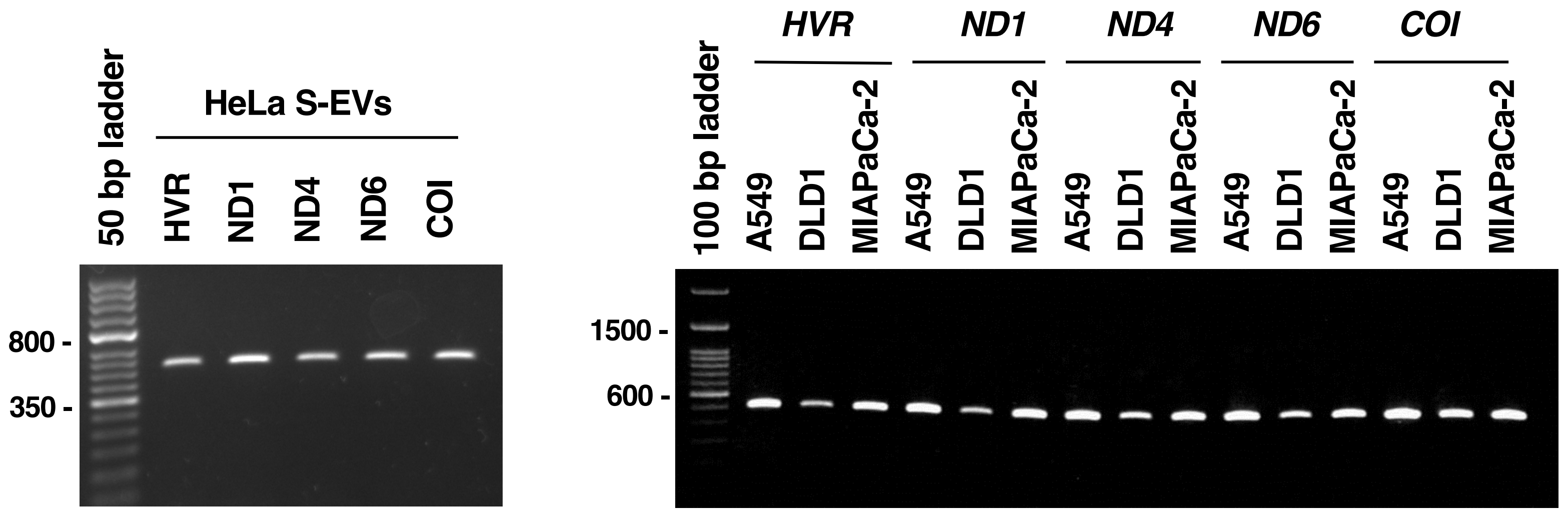

Fig. S4. PCR analysis of the presence of mtDNA in S-EVs isolated from various human cancer cell lines. S-EVs were isolated from the conditioned medium of HeLa cells, A549 cells, DLD1 cells and MIAPaCa-2 cells and subjected to PCR amplification of various mtDNA genes.

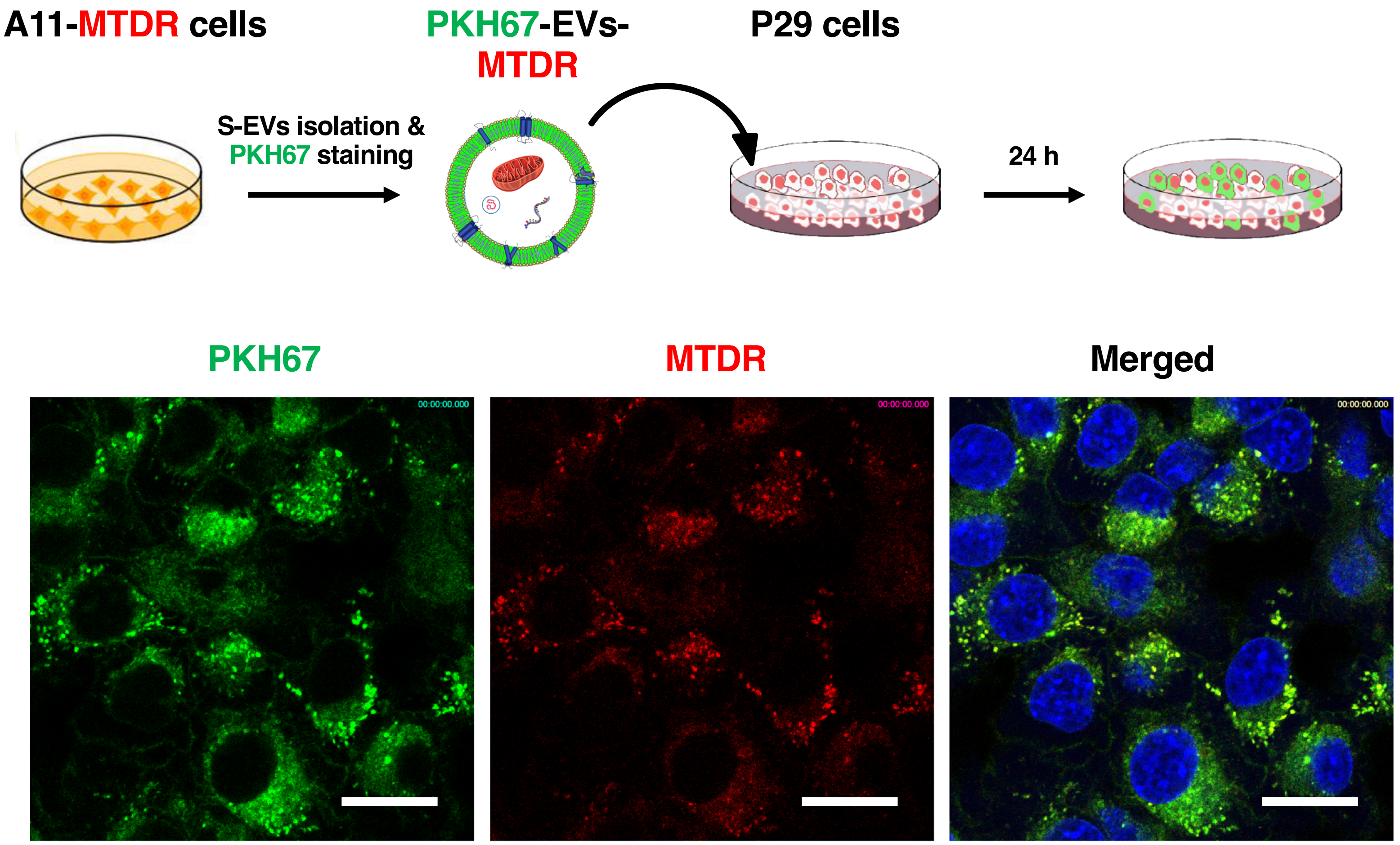

Fig. S5. Incorporation of A11 S-EVs into P29 cells. S-EVs isolated from the conditioned media of A11-MTDR cells were stained with PKH67, resulting in red and green two-colored PKH-67-positive S-EVs. They were then added to P29 cells and incubated for 24 h. Note that two-colored S-EVs are within P29 cells. Scale bars: 20 μm.

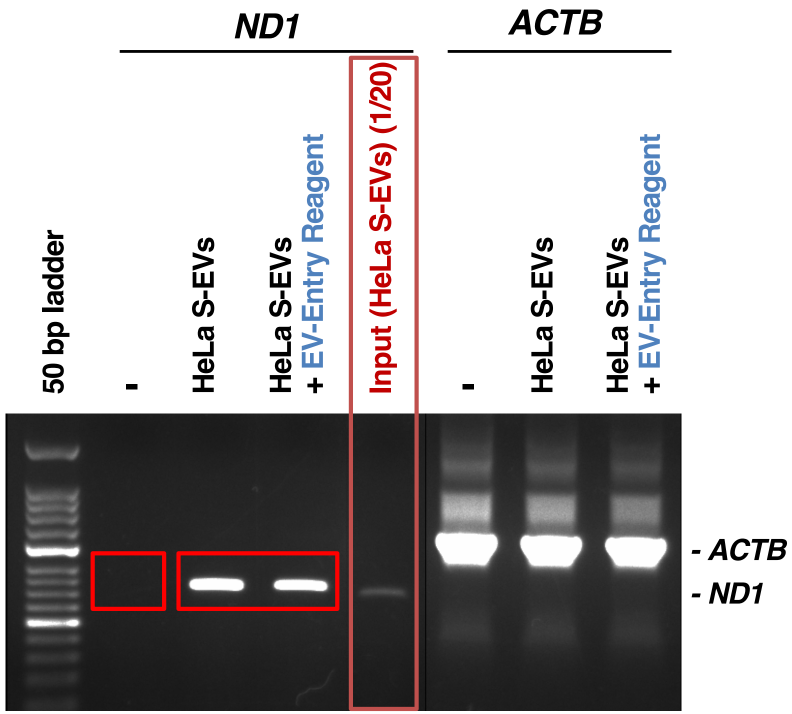

Fig. S6. S-EV-mediated mtDNA transfer to ρ^0HeLa cells. S-EVs isolated from the conditioned media of HeLa cells were incubated with ρ^0HeLa cells in the presence or absence of EV-Entry reagent for 2 days. The cells were detached from the dishes by trypsinization and washed extensively with PBS. DNA was isolated and then subjected to PCR amplification of *ND1* and *ACTB* (β-actin). The primers for *ND1* were the same as those described in Fig. S4. The primers for *ACTB* were used as a loading control. As an input, one-twentieth of the amount of S-EVs added to ρ^0HeLa cells was also subjected to PCR analysis.

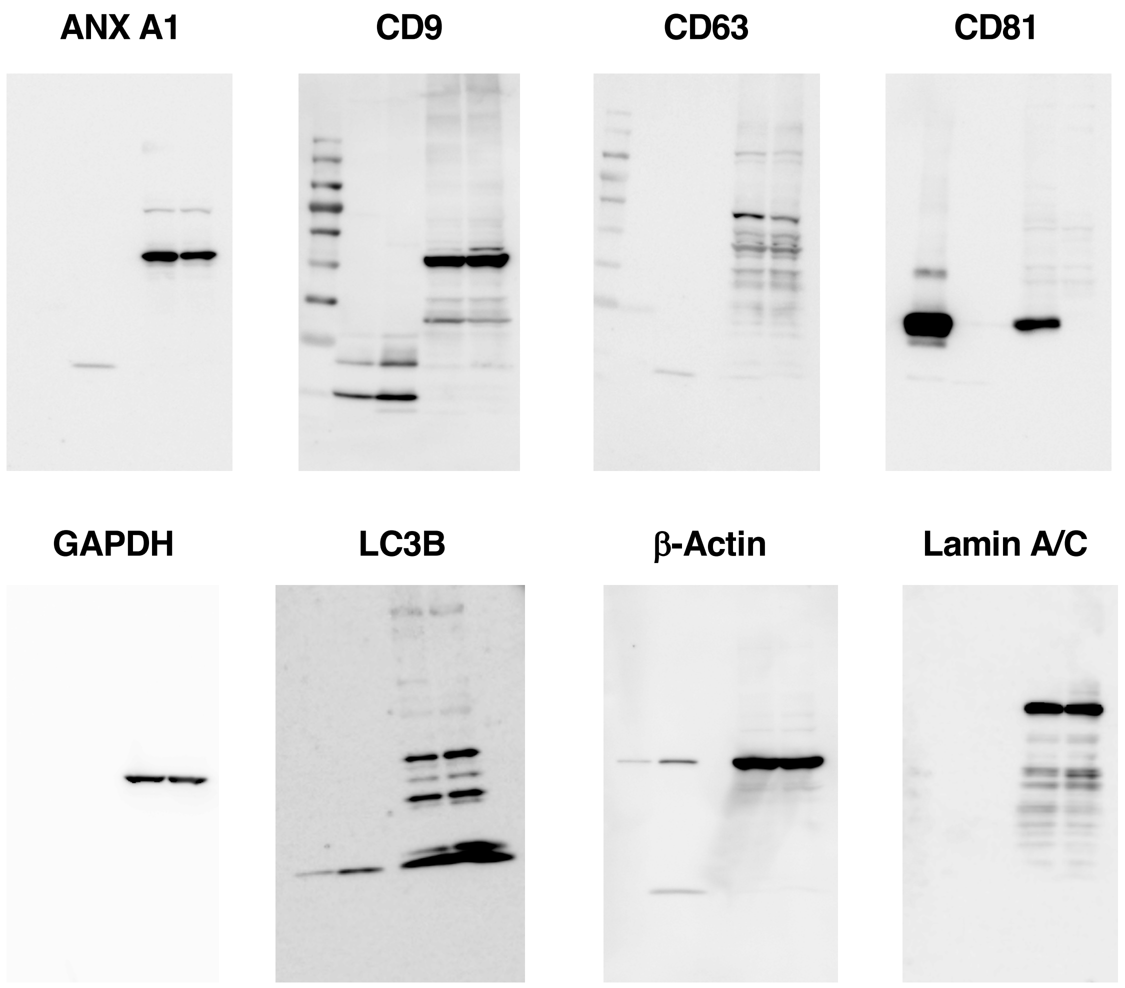

Fig. S7. Full-size images of Western blots.

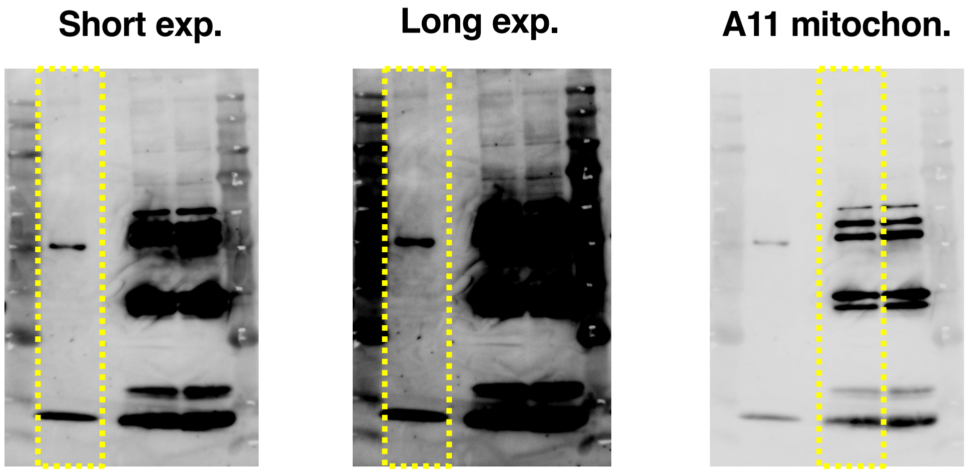

Fig. S8. Full-size images of Western blots. The yellow dotted line indicates the cropped region.
